# The Differential Absorption of a Series of P-Glycoprotein Substrates in Isolated Perfused Lungs from Mdr1a/1b Genetic Knockout Mice can be Attributed to Distinct Physico-Chemical Properties: an Insight into Predicting Transporter-Mediated, Pulmonary Specific Disposition

**DOI:** 10.1007/s11095-017-2220-5

**Published:** 2017-07-12

**Authors:** Daniel F. Price, Chris N. Luscombe, Peter J. Eddershaw, Chris D. Edwards, Mark Gumbleton

**Affiliations:** 1 Cardiff School of Pharmacy & Pharmaceutical Sciences, Cardiff University, King Edward VII Avenue, Cardiff, CF10 3NB UK; 2 GlaxoSmithKline Medicines Research Centre, Stevenage, Hertfordshire, UK

**Keywords:** efflux, isolated perfused lung, lung, MDR, *Mdr*, physico-chemical, pulmonary absorption, P-glycoprotein (P-gp), QSAR, transporter

## Abstract

**Purpose:**

To examine if pulmonary P-glycoprotein (P-gp) is functional in an intact lung; impeding the pulmonary absorption and increasing lung retention of P-gp substrates administered into the airways. Using calculated physico-chemical properties alone build a predictive Quantitative Structure-Activity Relationship (QSAR) model distinguishing whether a substrate’s pulmonary absorption would be limited by P-gp or not.

**Methods:**

A panel of 18 P-gp substrates were administered into the airways of an isolated perfused mouse lung (IPML) model derived from *Mdr1a*/*Mdr1b* knockout mice. Parallel intestinal absorption studies were performed. Substrate physico-chemical profiling was undertaken. Using multivariate analysis a QSAR model was established.

**Results:**

A subset of P-gp substrates (10/18) displayed pulmonary kinetics influenced by lung P-gp. These substrates possessed distinct physico-chemical properties to those P-gp substrates unaffected by P-gp (8/18). Differential outcomes were not related to different intrinsic P-gp transporter kinetics. In the lung, in contrast to intestine, a higher degree of non-polar character is required of a P-gp substrate before the net effects of efflux become evident. The QSAR predictive model was applied to 129 substrates including eight marketed inhaled drugs, all these inhaled drugs were predicted to display P-gp dependent pulmonary disposition.

**Conclusions:**

Lung P-gp can affect the pulmonary kinetics of a subset of P-gp substrates. Physico-chemical relationships determining the significance of P-gp to absorption in the lung are different to those operative in the intestine. Our QSAR framework may assist profiling of inhaled drug discovery candidates that are also P-gp substrates. The potential for P-gp mediated pulmonary disposition exists in the clinic.

**Electronic supplementary material:**

The online version of this article (doi:10.1007/s11095-017-2220-5) contains supplementary material, which is available to authorized users.

## Introduction

Alongside passive processes, active or facilitative transporters may govern the permeability of biological barriers to drugs. Following oral inhalation of drug, lung epithelial transporters may impact upon a drug’s intra-luminal residence time and delivery to pharmacological targets within the submucosal compartment as well as absorption to the systemic circulation. While there is increasing awareness of the range and pattern of expression of transporters within lung tissue (reviewed in (1,2)) the functional significance of transporters upon pulmonary drug disposition is however less well understood. In particular, given the extent of knowledge within other barriers comparatively little is known about the functional significance of P-glycoprotein (P-gp) within the lung.

Evidence for P-gp expression within whole lung is available at both the mRNA and protein level in humans (MDR1 gene) and rodents (*Mdr1a* and *Mdr1b* genes); in rodent lungs the *Mdr1b* transcript appears to be expressed in greater abundance than the *Mdr1a* transcript (3,4). Whether by PCR (4,5) or microarray (3) methods the lung displays relatively lower levels of P-gp mRNA expression compared to other biological barriers such as the ileum. However, the lung possesses significant cellular heterogeneity and it is the P-gp expression within lung epithelium itself, as well as potentially within intra-luminal macrophage populations and pulmonary capillary endothelium, which can be expected to be the most impactful for the pulmonary disposition of inhaled drug. Correspondingly, within fully intact lung tissue P-gp protein expression is recognised at the luminal surface of bronchial/bronchiolar epithelium (4,6–9) and within alveolar epithelium (4). Further, there are a number of in-vitro studies using alveolar and bronchial primary epithelial cells (4,10), or continuous lung epithelial cell lines (2,11,12), corroborating P-gp protein expression and reporting varying extents of polarised transport functionality. Nevertheless, the impact of P-gp upon the overall pulmonary absorption and disposition of airways administered drugs is poorly understood, and requires investigation within lung models where tissue architecture and the parallel processes of passive and active clearance mechanisms from the airways are to a large extent preserved.

In 2003 as part of a broader QSAR pulmonary absorption investigation Tronde *et al*. (13) reported upon the extent of pulmonary absorption of the P-gp substrates losartan and talinolol, respectively, 92% (t^1^/_2absorption_ 5 min) and 81% (t^1^/_2absorption_ 17 min) following their administration as nebulised solutions into the airways of anaesthetised rats. While the study was not explicitly addressing efflux transporter issues it led the authors to speculate that such high absorption suggests P-gp may have little, if any, influence on the pulmonary transport of P-gp substrates. Although, as acknowledged by the authors, the drug solutions were administered at mM concentrations (3.8 and 2.8 mM, respectively, for losartan and talinolol) which may have saturated efflux transporter activity. In the few studies undertaken using intact lung models that have explicitly addressed the impact of P-gp upon the absorption of substrates from the airways discordant conclusions exist. In 2008 Manford *et al*. (14) reported the pulmonary absorption of the P-gp substrate digoxin to remain unchanged in CF-1 mice which display a spontaneous *Mdr1a* knockout phenotype, although these mice retain expression of the *Mdr1b* gene. However, the same group also showed in an isolated perfused rat lung (IPRL) model that co-administration into the airways of a P-gp inhibitor, GF120918, had no effect upon the pulmonary absorption of digoxin (11). Concurrently a 2008 report by Francombe *et al*. (15), also using an IPRL model, showed the pulmonary absorption of the P-gp substrate rhodamine-123 (Rh-123) to be significantly increased by the co-administration of GF120918. In a follow-up study this discordance between digoxin and Rh-123 outcomes was explicitly examined in both IPRL and isolated perfused mouse lung (IPML) models (16). Again using GF120918 to modulate P-gp activity the pulmonary absorption of Rh-123 was increased by P-gp inhibition, whereas that for digoxin remained unaffected; the same study also showed discordance of the effect of P-gp upon the pulmonary absorption of the P-gp substrates saquinavir (unaffected) and loperamide (affected).

The airway to pulmonary vasculature transport of P-gp substrates will, as in other barriers and notably well documented for the intestine, reflect a balance between a substrate’s physico-chemical properties driving membrane affinity and transmembrane movement rate *versus* the substrate’s exposure and engagement with P-gp within the membrane. However, given the lung’s recognised permeability for hydrophilic molecules we hypothesised that in the lung, in contrast to the intestine, a higher degree of non-polar character is required of any P-gp substrate before the net effects of P-gp efflux become evident. Here we report current work exploring this question by examining the pulmonary absorption of an 18-member panel of P-gp substrates within an IPML model using lungs from genetic *Mdr1a*/*Mdr1b* (−/−) double knockout mice. The intestinal permeability of the panel of substrates  was also studied using ileal segments from the same knockout animals. P-gp binding studies and physico-chemical profiling was undertaken to build an orthogonal PLS Discriminant Analysis (oPLS-DA) model based solely upon calculated physico-chemical descriptors for the substrates. This oPLS-DA model displayed capacity to predict whether P-gp would impact upon the absorption of airway administered substrates. In this work we have identified a distinct relationship between substrate physico-chemical properties and P-gp efflux operative in the lung. The work advances our understanding of the impact of P-gp upon drug absorption from airways with potential relevance to the clinical setting, and provides a QSAR framework of physico-chemical characteristics that may assist the profiling of inhaled drug discovery candidates that are also P-gp substrates.

## Materials and Methods

### Materials

Radiolabelled [^14^C]- or [^3^H]-mannitol and the P-gp substrate [^3^H]-digoxin were purchased from American RadioChemicals (St. Louis, MO). The P-gp substrates GSK-1,-2 and −3, and indacaterol were supplied by GlaxoSmithKline (Stevenage, UK). Unlabelled P-gp substrates also contributing to the test panel of compounds included: acrivastine, erythromycin, mitoxantrone, monensin, puromycin, saquinavir, chloroquine, colchicine, domperidone, eletriptan, rhodamine-123 (Rh-123), salbutamol and salmeterol, all obtained from Sigma Aldrich (Poole, UK). All other chemicals and solvents were obtained from Sigma-Aldrich or Fisher Scientific at the highest available purities.

### Animals

Male wildtype CD-1 mice were obtained from Harlan, UK. Male *Mdr1a*(−/−)/*Mdr1b*(−/−) knockout mice (FVB strain) were from Taconic (USA) and cross bred in-house (Cardiff) with wildtype *Mdr1a*(+/+)/*Mdr1b*(+/+) female B6 strain mice. Genotyping of F2-F5 generations allowed the selection of homozygous breeding pairs of FVB/B6 *Mdr1a*(−/−)/*Mdr1b*(−/−) mice and establishment of the FVB/B6 knockout colony. Genotyping involved extraction of genomic DNA from mouse tissue (DNeasy Mini Kit, Qiagen, Hilden, Germany) according to manufacturer’s protocol. Genomic DNA samples (2 ng) were amplified by PCR using HotStarTaq DNA Polymerase kit (Qiagen) with a recipe of: 2.5 μL 10x PCR buffer (final MgCl_2_ of 1.5 mM); 0.5 μL 10 mM dNTPs; 0.125 μL DNA polymerase; 0.5 μL 10 μM primer stock, and nuclease free water (Ambion, TX, USA) to a 25 μL final volume. The reaction was maintained at 95°C for 15 min followed by thermal cycling (35 cycles: 94°C for 1 min, 55 or 60°C for 1 min, 72°C for 1 min with a final extension of 72°C for 10 min). DNA fragments were separated by gel electrophoresis (1.25% agarose, 80 V (6.5 V/cm) for 45 min) and visualised under UV light using EtBr staining with a 1 kb + DNA ladder (New England Biolabs).

Primer sequences for the amplification of *Mdr1a* were provided by Taconic, primer sequences for the amplification of *Mdr1b* were designed in-house against the murine *Mdr1b* sequence (GenBank ID 18669) and the sequence of the disruptive neomycin cassette inserted to the *Mdr1b* gene sequence (Supplementary Table S1).

At experimentation all mice weighed 25–35 g and were housed under barrier conditions with 12 h light-dark cycles at 21°C and 45–60% humidity, and with access to food and water *ad Libitum*. Isolated organ experiments conformed to schedule 1 with animals euthanised by high dose i.p. injection of sodium pentobarbital (Euthatal®) prior to surgery. All other animal studies were ethically reviewed and carried out in accordance with Animals (Scientific Procedures) Act 1986 and the GSK Policy on the Care, Welfare and Treatment of Animals.

### Isolated Perfused Mouse Lung (IPML)

The pulmonary transport of the test panel of P-gp substrates was studied using an IPML model as described previously (16). Male mice (30 ± 3 g) were euthanised and the trachea exposed and cannulated (mouse tracheal cannula of L - 20 mm, OD −1.3 mm, ID - 1.0 mm; Harvard Apparatus, UK) with insertion of cannula to a distance of 2–3 mm short of the first bifurcation. Abdominal and thoracic cavities were exposed and the pulmonary artery catheterised and perfused (recirculating mode at a rate of 1 mL.min^−1^ using a peristaltic pump, Munipuls 3, Gilson, USA) with oxygenated, 95% O_2_ / 5% CO_2_, 37°C Krebs-Henseleit buffer supplemented with 4% *w*/*v* BSA. The atria and the lower half of the heart were cut to allow the free flow of perfusate. The lungs were removed and suspended vertically in a custom water-jacketed glass thorax (Radleys, Essex, UK) maintained at 37°C. Following a 5 min equilibration the lungs were then ventilated throughout the experiment (0.3 mL of air, 70 breaths.min^−1^, mechanical ventilator 501,718, Harvard, UK).

Compounds were administered into the IPML airway in a volume of 25 μL (PBS pH 7.4) using a gas-tight stoplock Hamilton syringe (1700 series) and involving the simultaneous co-administration of a bolus volume of 250 μL air helping to both inflate the lungs and provide more extensive and reproducible distribution of drug to the lung lobes and periphery**.** This dosing method results in a reproducible >94% deposition of dose to the lung lobes with the remaining (ca. 6%) deposited in the tracheal cannula and trachea/major bronchi. The individual lobar deposition of compound (expressed as a % deposited dose) has also been shown to be proportional to the lobar mass (16). In each experiment IPML viability was assessed over a 45 min window, confirmed by monitoring of the absorption of the hydrophilic solute, mannitol, and post-experiment measurement of the wet/dry weight ratio of the lungs. IPML experiments were discarded (typically 1 in 10 preparations) where visible or quantitative evidence of pulmonary oedema within the lungs was observed. Typically wet:dry weight ratios averaged 3.32 ± 0.34 in freshly isolated lungs compared to 3.23 ± 0.27 for lungs used in IPML experiments. Similarly IPML experiments were discarded where co-instilled radiolabelled mannitol absorption was >65% of deposited dose over the 30 min experimental period.

To avoid differential solvent effects, and irrespective of the physico-chemical properties of each of the 18 P-gp substrates within the panel, the 25 μL dose solutions administered into the IPML airways all included a final concentration of 0.1% DMSO. Non-radiolabelled P-gp substrates were all administered at a dose of 1.25 nmoles (based on free drug) and equating to a concentration in the 25 μL dose solution of 50 μM. Radiolabelled [^3^H]-digoxin was administered at a dose of 16 pmoles equating to a concentration of 0.64 μM. Included in all dose solutions for every experiment was 0.22 μM [^3^H]-mannitol as the hydrophilic paracellular marker used as an indicator of intrinsic lung barrier integrity; [^14^C]-mannitol was used in experiments involving [^3^H]-digoxin. All compounds were confirmed to have negligible binding to the tubing and glassware used in the IPML setup.

### Bioanalysis

Following dosing of compound into the IPML airways serial samples (250 μL) were collected for bioanalysis from the 10 mL recirculating perfusate reservoir. Each sample collected from the reservoir was replaced with an equal volume of fresh warmed, oxygenated perfusate. Upon termination of the experiment the dose solutions, apparatus washings and perfusate samples were collected for mass balance calculations enabling determination of the actual total dose delivered to the lungs.

Radioactive compounds were directly analysed by liquid scintillation counting. Volumes of 50 μL perfusate samples were mixed with 3 mL of Scintisafe 3 Liquid Scintillation Fluid (Fisher, UK) and transferred to vials for scintillation counting using a TriCarb 2900TR (Packard Bioscience, USA). Analysis for non-radiolabelled substrates involved deproteinisation by precipitation with 2x volume of ice-cold acetonitrile (ACN) or methanol (MeOH) to the sample followed by centrifugation (20,000 g 15 min at 4°C) with the resulting protein-free supernatant undergoing analysis by LC-MS or LC-MS/MS ESI + ve ionisation (Thermo Finnigan LCQ Classic). Samples were analysed using a C-18 HPLC column (Kromasil, 100 Å pore, 3.5 μm particle size) at 30°C with a mobile phase (0.2 mL/min) comprising a various gradients of solvent A: MeOH or ACN, and solvent B: H20 + 0.1% Formic Acid; MS/MS was used for domperidone, mitoxantrone, monensin, saquinavir. Standard curves for all compounds were prepared in perfusate and included as a reference pre-dose perfusate that had been passed through the IPML. At a concentration of 3 ng/mL (the set LLQ) all analytes displayed a quantitation accuracy within ±13% and a precision within 10%, but for most analytes these parameters were considerably smaller than this (Supplementary Table S2).

After accounting for the mass of compound removed at each sample point, plots of the cumulative ‘%deposited dose absorbed’ *versus* ‘time’ were generated and subject to nonlinear regression analysis according to a first-order absorption one-compartment accumulating model (Eq. 1) to generate estimates for the extent (F) and rate (Ka) of absorption from the IPML airways to the recirculating perfusate. For each compound preliminary studies had confirmed negligible loss from the recirculating perfusate to lung tissue or to the IPML tubing or glassware.

1 $$ \% Dose\  absorbed={\left({100}^{\ast } F\right)}^{\ast}\left(1-{e}^{- Ka\ast t}\right) $$

The data was also subject to standard non-compartmental analysis, specifically Area Under Curve (AUC_0–30 min_) of ‘%deposited dose absorbed’ *versus* ‘time’ (units: %Dose.min) and reflecting for the exclusively accumulating perfusate ‘compartment’ the exposure arising from the IPML absorption process alone.

## Composition of P-Gp Substrate Test Compound Data Set

The impact of a deficiency of P-gp upon the pulmonary absorption of the P-gp substrates digoxin (14) and rhodamine-123 (15,16) had previously been explored in isolated perfused lung models, as such these compounds represented the respective archetypes for Group A and Group B substrates (see below). The remainder of the 18 member P-gp substrate panel were selected on the basis of: (i) providing a diverse spectrum of physico-chemical properties; (ii) inclusion of inhaled compounds; (iii) availability of sensitive LC-MS/MS assays, and (iv) evidence that all had demonstrated P-gp mediated efflux within an accepted published model (17,18) or within in-house models for the GSK compounds (unpublished data).

The outcomes of the IPML experiments allowed categorisation of the test panel into Group A compounds whose absorption was not affected in the IPML by /*1b* knockout, and Group B compounds (Table I) whose absorption was increased in the IPML by *Mdr1a*/*1b* knockout; this categorisation was then used in describing the outcomes from all other experimental approaches.

**Table I:** Computed Physico-chemical parameters for Group A and Group B compounds

		cLogD (7.4)	cLogP	Abraham Acidity (>0.75)	Abraham Basicity (>3)	H bond donor (acidity)	H bond acceptor (basicity)	H bond total	Rotatable bond count	Abraham Volume	Abraham Polarizability (>3)	Polar Surface Area (Å)^2^	MW	Abraham Molar Refractive Index
GROUP A	Acrivastine	2.03	4.55	0.59	1.50	1	4	5	6	2.81	2.07	53	348	2.10
	Digoxin	0.90	0.90	1.72	4.62	6	14	20	13	5.75	5.34	203	780	3.20
	Erythromycin	1.20	1.90	1.02	4.71	5	14	19	12	5.77	3.55	194	733	1.97
	GSK1	3.07	3.75	1.08	2.67	10	3	13	7	3.83	5.28	130	571	3.83
	Mitoxantrone	1.00	1.55	0.52	2.88	8	10	18	16	3.29	2.06	163	444	3.31
	Monensin	0.96	4.01	1.49	3.55	4	11	15	13	5.24	2.44	153	670	1.61
	Puromycin	0.97	1.30	0.51	3.56	5	12	17	11	3.42	3.58	164	471	3.38
	Saquinavir	3.60	4.03	1.61	4.68	6	11	17	14	5.89	6.30	167	670	4.25
GROUP B	Chloroquine	1.59	4.41	0.20	1.59	1	3	4	8	2.63	1.73	28	319	1.91
	Colchicine	1.07	1.07	0.37	1.96	1	7	8	5	2.99	2.96	87	399	2.11
	Domperidone	2.93	4.05	0.65	2.23	2	7	9	5	3.06	3.09	79	425	3.81
	Eletriptan	0.23	2.98	0.003	1.84	1	4	5	6	2.93	3.17	53	382	2.74
	GSK2	2.64	3.28	0.83	2.56	7	2	9	6	3.76	3.78	100	495	3.76
	GSK3	3.89	3.11	0.003	0.80	3	0	3	9	3.88	2.36	33	466	3.88
	Indacaterol	2.42	-1.71	0.54	2.06	5	1	6	4	3.09	2.54	59	393	3.09
	Rh-123	4.01	4.01	0.50	1.21	4	5	9	5	2.56	2.90	90	344	3.27
	Salbutamol	-1.44	0.70	1.08	1.91	4	4	8	8	1.98	1.41	73	239	1.41
	Salmeterol	1.15	3.07	1.08	2.11	4	5	9	19	3.49	2.11	82	416	2.07

### ABC Transporter Membrane ATPase Assay

The Gentest ATPase assay (Corning, Germany) was used to investigate the binding kinetics of the panel of P-gp substrates against membranes constituted to display human MDR1, mouse *Mdr1a*, mouse *Mdr1b* and mouse *Bcrp*. The assays measure the accumulation of inorganic phosphate (absorbance at 800 nm) produced in the breakdown of ATP to ADP. The assays are an indirect measure of ABC transporter interactions and do not distinguish between substrates or inhibitors. The assays were used in accordance with the manufacturer’s protocol to calculate (GraphPad Prism) the Michaelis-Menten parameters K_m_ and V_MAX_. For the assay test compounds were serially diluted to produce an assay concentration range of 0–300 μM with the kinetics of ATP turnover examined over 30 min at 37°C.

### Computational Physico-Chemical Properties

The primary physico-chemical properties: cLogP, cLogD, H-bond donors, H-bond acceptors, rotatable bond count, molecular volume and molecular weight were determined for the test panel using the online ACD/Labs software with the SMILES string for each substrate as the input. Polar surface area and solvent accessible surface area were calculated using ChemBio3D Ultra 11.0. The Abraham solvation descriptors were calculated using an additive model based on molecular fragments as previously described (19).

### Immobilised Artificial Membrane (IAM) Chromatography

IAM chromatography provided an experimental measure of substrate membrane affinity and phospholipid interaction. The IAM column (Regis Technologies Inc., Hichrom, Reading, UK; IAM.PC.DD2 10 cm x 4.6 mm ID column) was run under isocratic eluting conditions with the retention of all compounds determined under three mobile phase conditions: 10%, 20% and 30% acetonitrile in water, and from which the theoretical retention time in 100% water was calculated by linear regression. LogK^IAM^ values were calculated according to Eq. 2:

2 $$ Log{K}^{IAM}= Log\left[\frac{\left({T}_r-{T}_0\right)}{T_0}\right] $$

where T_r_ and T_0_ are respectively the retention times of the substrate in 100% water and of the unretained compound, i.e. eluting within the solvent front of 75 s in all experiments.

### Multi-Lamellar Liposome Vesicle (MLV) Partitioning

MLVs were formed as previously described (20). Briefly, solutions of 8 mg phosphatidylcholine and 2 mg phosphatidylglycerol in 10 mL chloroform were completely dried to a lipid film (rotation under vacuum). MLVs were formed by the addition of 1 mL of 25 mM HEPES-Tris buffer (pH 7.5) with gentle shaking for 4 h. The suspension was then centrifuged at 6000 g for 15 min to pellet the vesicles and the supernatant was removed. The MLVs were resuspended in 10 mL of PBS (pH 7.4) and stored at 4°C under nitrogen gas overnight before partitioning experiments were performed. The average lipid yield in the formed MLVs was estimated by freeze drying and was 46 ± 8% (a value consistent with Eytan *et al*. (20) studying MLV partition of P-gp substrates) and generating an MLV suspension containing 10–15 μM of phospholipid.

For partitioning experiments the MLV suspension was diluted 1000-fold in PBS (pH 7.4) with 990 μL transferred to a microfuge tube into which 1 μg (10 μL) of P-gp substrate was added. These preparations were then incubated at 37°C for 4 h under shaking conditions to allow equilibration of compound between the lipid and aqueous fractions. The MLVs were then pelleted and 100 μL of supernatant removed for analysis by radiometric or LC-MS/MS methods as described above. Partitioning into the MLVs was determined according to Eq. 3:

3 $$ MLV\  partitioning=\left[{C}_0-{C}_S\right]/{C}_0 $$

where C_0_ is the initial compound concentration (1 μg/mL) and C_S_ is the compound concentration present in the aqueous supernatant at the end of 4 h incubation.

### Ussing Chamber Intestinal Permeability

Following isolation of the lung tissue for IPML experiments the same animals were used to obtain *Mdr1a*/*1b* (+/+) and *Mdr1a*/*1b* (−/−) intestinal tissue. The mouse Ussing chamber experiments were performed as previously described by others (21–23) including retention of the seromusculature layer whose removal can easily damage the mouse intestinal mucosa. The presence of the seromusculature layer itself also preserves the intramural neuromuscular connections and is reported not to impact to any significant extent upon the permeability of hydrophilic or hydrophobic drug solutes (21).

The distal third (distal jejunum and ileum) of the mouse small intestine was isolated and flushed with protein-free Krebs-Henseleit buffer (pH 7.4) to remove faecal matter, after which 1 cm length segments were cut (avoiding any visible Peyer’s patches), opened longitudinally and mounted within a vertical tissue diffusion chamber system (Harvard). The bathing fluid for both mucosal and serosal surfaces (3 mL volume in each chamber) comprised oxygenated Krebs-Henseleit buffer maintained at 37°C (heating block) under stirred conditions (gas flow manifold). The tissue was allowed to equilibrate for 15 min after which transepithelial electrical resistance (TEER) was recorded (gas flow temporarily halted) using NaviCyte Ag/AgCl electrodes and an Epithelial voltage clamp (Harvard). Any intestinal segments presenting an initial TEER of lower than 80 Ω.cm^2^ were discarded prior to the experiment, likewise any segments whose TEER decreased by more than 10% over the course of the experiment were excluded. The viability of such tissues preparations has been shown to extend to 4 h.

The concentrations of the P-gp substrates dosed into the Ussing chamber experiments were matched to those used in the IPML experiments, e.g. for the non-radiolabelled substrates the final concentration in the mucosal barrier chamber was 50 μM in Krebs-Henseleit buffer. Following application of the test compounds, serial samples (1000 μL) for analysis were collected over a 180 min period from the serosal chamber with the removed sample replaced with an equal volume of fresh warmed and oxygenated buffer. Samples were subject to radiochemical or LC-MS/MS bioanalysis as for the IPML studies. Thereafter permeability coefficients were determined according to Eq. 4:

4 $$ \rho =\left[\frac{dM}{dt}\right]/\left[{A}^{\ast }{C}_0\right] $$

where dM/dt is the rate of transport observed across the 180 min experiment, A is the surface area of the intestinal segment exposed in the experiment (0.12 cm^2^) and C_0_ is the initial donor concentration of the compound.

### Development of a QSAR Classification Model

To evaluate which physico-chemical properties were the main drivers associated in determining whether compounds display Group A absorption properties in the IPML (absorption not affected by *Mdr1a*/*1b* knockout) or Group B properties (absorption increased in the IPML by *Mdr1a*/*1b* knockout) the physico-chemical descriptors for the 18 substrate molecules were used to build an orthogonal PLS Discriminant Analysis (oPLS-DA) model (SIMCA v13.03, Umetrics AB). Thirteen physico-chemical properties (Table I) that generally describe lipophilicity, hydrogen bonding, size, shape, charge and atom polarisability properties were calculated from the molecular structures and used as the input descriptors of the model.

### Statistical Analyses

The following statistical approaches were used in either GraphPad PRISM or SPSS, and are described within the individual Figure and Table legends: T-test for independent samples; One-way AVOVA and Tukey post-hoc for comparisons of each mean with every other mean or Dunnett post-hoc comparing every mean to control; Pearson Correlation coefficient and for ranked correlations Spearman Correlation coefficient, both two-tailed; Power Analysis for sample sizes. Nonlinear regression was undertaken by WinNONLIN, and PLS Discriminant Analysis (oPLS-DA) undertaken using SIMCA.

## Results

### Differential Absorption of P-Gp Substrates in the IPML: Impact of P-Gp Knockout

We established a colony of FVB/B6 *Mdr1a*/*1b* (−/−) knockout mice where the complete disruption of both *Mdr1a* and *Mdr1b* genes was confirmed by multiplex PCR (Supplementary Fig. S1A). The homozygous FVB/B6 *Mdr1a*/*1b*(−/−) knockout and strain matched wildtype *Mdr1a*/*1b*(+/+) mice were used in IPML preparations as previously described (16), where the consistency of airway dose and lobar pattern deposition of substrate as well as the viability of the tissue barrier, had previously been confirmed.

The disruption of the *Mdr1a*/*1b* genes did not affect pulmonary barrier permeability *per se* as evidenced by the airway to perfusate absorption of the co-instilled paracellular probe mannitol, the absorption of which remained unchanged between the *Mdr1a*/*1b*(−/−) and *Mdr1a*/*1b*(+/+) FVB/B6 groups (respectively, 43.2 ± 10.2% and 44.3 ± 9.4% of the deposited dose absorbed by 30 min). Mannitol permeation in the lungs of the FVB/B6 mice was also comparable to that for wildtype CD1 albino mice (Supplementary Fig. S1B). We noted no differential effects of any of the compounds in the panel to alter mannitol permeability.

**Fig. 1:**
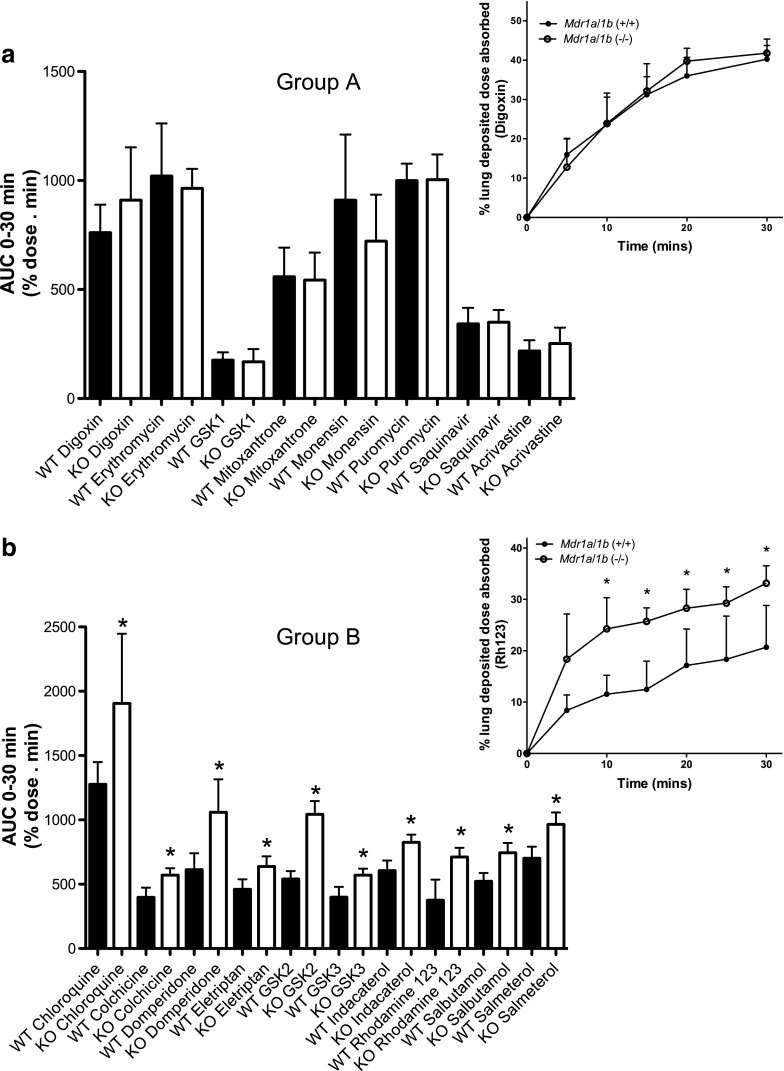
Impact of *Mdr1a*/*1b* (−/−) knockout upon pulmonary absorption of airway dosed P-glycoprotein substrates: (**a**)shows the cumulative absorption (defined by AUC_0–30 min_ calculated on drug levels in the IPML recirculating perfusate) for Group A compounds in IPML preparations from *Mdr1a*/*1b*(−/−) knockout mice (KO, unfilled bar) and wild-type *Mdr1a*/*1b*(+/+) mice (WT, filled bars). Insert shows IPML absorption profile for digoxin, the archetype Group A compound; (**b**)shows the cumulative absorption of Group B compounds in IPML preparations from *Mdr1a*/*1b*(−/−) knockout mice (KO, unfilled bar) and wild-type *Mdr1a*/*1b*(+/+) mice (WT, filled bars). Insert shows IPML absorption profile for Rh-123, the archetype Group B compound. Data represent mean ± S.D., *n* = 4 to 6 mice for each treatment arm. * indicates statistical difference at *P* < 0.05.

Figure 1a shows a plot of the perfusate AUC_0–30 min_ values for the eight ‘Group A compounds, defined as such by their absorption in the IPML remaining unaffected by *Mdr1a*/*1b* knockout, and where digoxin served as the archetype compound (Fig. 1a insert). Here the perfusate AUC_0–30 min_ parameter reflects absorption across the pulmonary barrier into what is a non-eliminating accumulating perfusate compartment. A similar plot is shown for the ten Group B compounds (Fig. 1b) where Rh-123 served as the archetype compound (Fig. 1b insert). Only for the Group B compounds did the deletion of *Mdr1a*/*1b* (−/−) result in significant increases (37% to 93%) in pulmonary absorption (*P*-values 0.003 to 0.027, Supplementary Table S3). The corresponding perfusate profiles for each of the Group A and Group B compounds are shown in Supplementary Figs. S2 to S5, where significant differences in the absorption profiles between the *Mdr1a*/*1b* (−/−) and *Mdr1a*/*1b* (+/+) mice were seen for the Group B compounds only.

The differential impact of *Mdr1a*/*1b* deletion between the two groups of compounds appeared unrelated to the extent of absorption per se*.* In *Mdr1a*/*1b* (+/+) lungs the average % dose absorbed was 26.3% (95% CI of 20.5–31.9%) for the Group A compounds, while for Group B compounds the respective value was 30.5% (18.9–42.3%) (*P* = 0.36). The observed extent of absorption for all 18 compounds as well as pharmacokinetic model estimates of bioavailability (F) are shown in Supplementary Table S4. While the pharmacokinetic model fit represented the observed data well with model estimates for ‘F’ showing low variance, the estimates for the first-order absorption rate constant, ‘Ka’, were less reliable (typically % CV of ca. 50%) and were not improved by applying different weighting to the model parameters or by more complex models. The greater variance in the Ka parameter probably reflected variability caused by the rapidly changing perfusate concentrations at the early time points combined with a limitation in collecting enough observations to accurately determine the initial rate.

### Comparative Interaction of Substrates with P-Gp Membranes

**Fig. 2:**
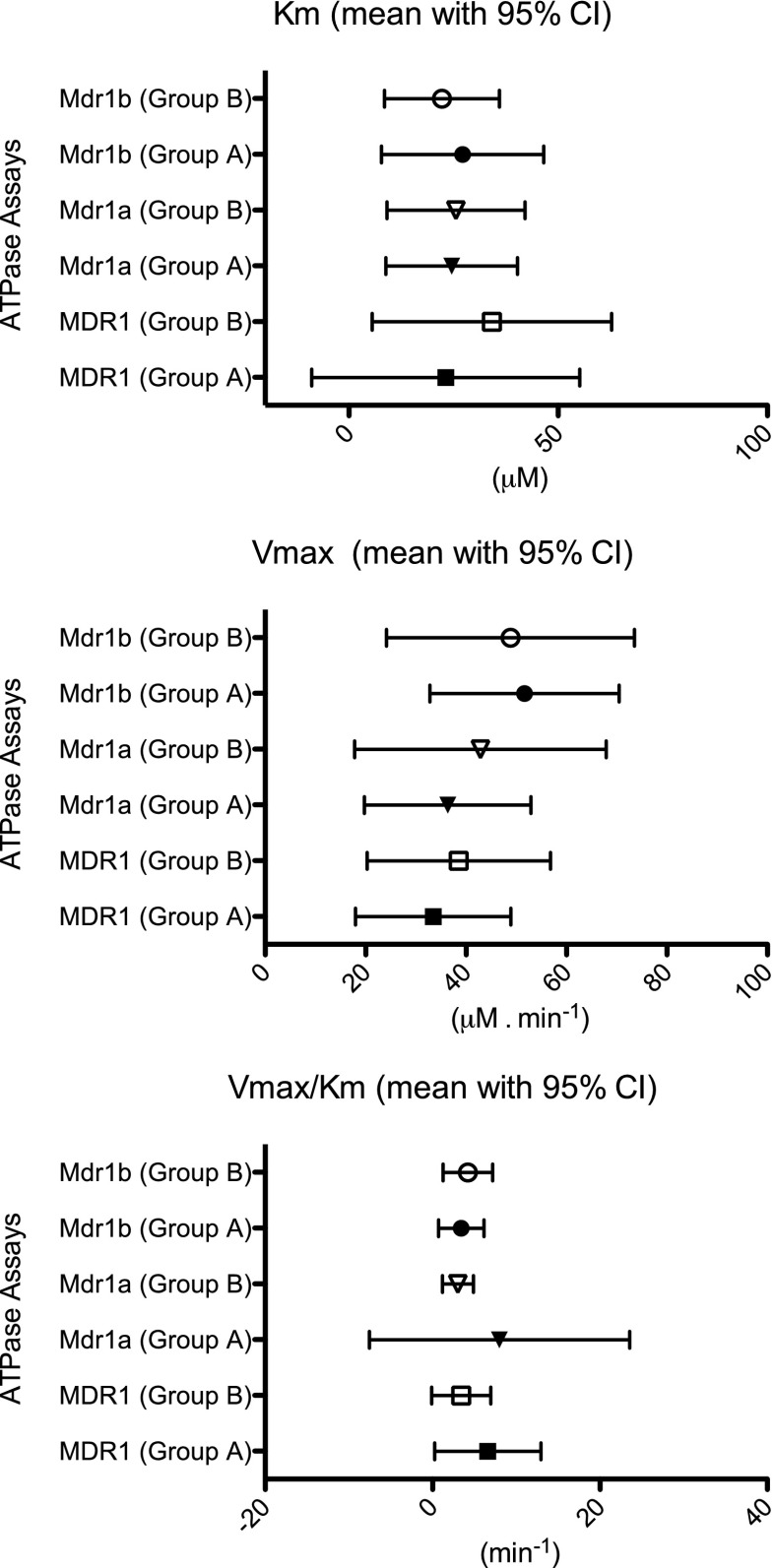
Binding interactions of P-glycoprotein substrates assessed against human MDR1, and rodent *Mdr1a* and *Mdr1b*: (**a**) comparative binding data for K_m_ (μM); (**b**) comparative binding data for V_max_ (μM . min^−1^); (**c**) comparative binding data for V_max_/K_m_ (min^−1^). Data represents mean with associated 5% to 95% confidence interval (CI) from three independent experiments, where in each experiment any substrate was examined in duplicate across a 1–300 μM concentration range.

All of the compounds used in the panel had previously been identified through functional studies to be P-gp substrates. Here we examined for differential P-gp binding kinetics within the panel of compounds using ATP assays in artificial membranes constituted by human (MDR1) and mouse (both *Mdr1a* and *Mdr1b*) P-gp. Not surprisingly the assays indicated all compounds to interact with P-gp with both the Km and Vmax data for the 18 compound panel showing significant correlations (*P* < 0.001, Pearson Correlation Coefficient) across the MDR1, *Mdr1a* and *Mdr1b* membranes (Supplementary Table S5). Importantly, no significant difference (*P* > 0.05) was found in any of the kinetic parameters between the Group A and the Group B substrates (Fig. 2). Additionally we studied the interactions of the compounds with mouse breast cancer resistance protein (*Bcrp*). In the current work we found the vast majority of the 18 compound panel not to generate ATP turnover in the mouse *Bcrp* membrane model (Supplementary Table S5). In addition to evidenced *Mdr1a*/*1b* interactions, only four of the compounds also showed evidence of interactions with *Bcrp*. Namely two Group A compounds - GSK1 and mitoxantrone, and two Group B compounds - chloroquine and Rh-123. Indeed it is for these very four compounds where some literature evidence (varying in strength) supports BCRP/*Bcrp* interactions, either as a substrate or inhibitor. An important point to make is that the ATP assays should not be viewed as having direct quantitative meaning to transport functionality in biological membranes, rather it is a way of ranking the kinetics of interactions for a subset of compounds against a given membrane model.

### Differential Physico-Chemical Properties of P-Gp Substrates

**Fig. 3:**
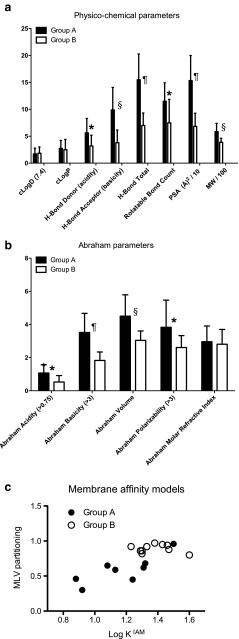
Physico-chemical and membrane affinity parameters for the P-glycoprotein substrates. (**a**) Computed primary physico-chemical parameters for Group A compounds (filled bars) *versus* Group B compounds (unfilled bars). (**b**) Corresponding computed Abraham physico-chemical descriptors; (**c**) Experimentally determined membrane affinity (Log K^IAM^) parameter for Group A and Group B compounds (Immobilised artificial membrane, IAM, chromatography) compared to liposome multi-lamellar vesicle partitioning (MLV partitioning). Compound specific data in Tables I and III. For 3A and 3B, * indicates statistical difference at *P* < 0.05, ^§^ indicates statistical difference at *P* < 0.01, ^¶^ indicates statistical difference at *P* < 0.001.

The discordance across our panel of compounds in respect to the net effect of P-gp upon IPML absorption may reflect distinct differences in the compounds’ passive membrane interactions. We first used computational approaches to determine the physico-chemical properties and Abraham solvation descriptors for each compound (Table I and Fig. 3a and b). The Group A compounds possessed a distinctly more polar character, the very characteristics of P-gp substrates that, at least in the intestine (24), would be indicative of substrates displaying a slower transmembrane movement rate and be subject (all other things being equal) to a greater net effect of P-gp efflux. To more fully define the capacity for hydrogen bonding interactions we used a fragment-based approach to calculate Abraham solvation descriptors (Fig. 3b). The Group A compounds showed significantly greater (*P* < 0.05) Abraham acidity and Abraham basicity values as well as Abraham polarizability, all indicative of a greater strength of hydrogen-bonding and a reduced affinity for biological membranes.

We next undertook IAM chromatography to experimentally quantify molecule affinity with phospholipid membranes; compounds showing a reduced extent of interaction with the phospholipid stationary phase possessing a lower LogK^IAM^ value. As P-gp substrates all of the compounds were retained on the IAM column, indicative of their capacity for phospholipid interactions, however, consistent with the computational descriptors the LogK^IAM^ values for the Group A compounds were significantly (*P* = 0.017) smaller than those for the Group B compounds (Table II). We also examined partitioning of the compounds within phosphatidylcholine and phosphatidylglycerol MLV liposomes, determining a partitioning parameter reflective of both initial membrane affinity of the compound and the compound’s transmembrane movement rate across the MLV structure (20). Consistent with the IAM chromatography the Group A compounds displayed a significantly (*P* = 0.0003) reduced partitioning compared to the Group B compounds (Table II). Not surprisingly these two methodologies are recognised to be correlative (25), and indeed the current work also showed them as such (Pearson Coefficient = 0.765; *P* = 0.0002) (Fig. 3c).

**Table II:** Experimental determination of membrane affinity by IAM column chromatography and membrane partitioning in multi-lamellar vesicle (MLV) liposomes. The 18 substrates are grouped into those molecules whose absorption in the IPML was unaffected by P-gp knockout (Group A) or those molecules whose absorption in the IPML was increased by P-gp knockout (Group B). Data represent the mean of four independent experiments. ^§^ indicates significant difference by unpaired T-test between Group A and B at *P* = 0.017 ^¶^ indicates significant difference by unpaired T-test between Group A and B at *P* = 0.0003

		LogK^IAM^	MLV Partitioning
GROUP A	Acrivastine	1.50	0.96
	Digoxin	0.92	0.30
	Erythromycin	0.88	0.46
	GSK1	1.08	0.65
	Mitoxantrone	1.13	0.59
	Monensin	1.32	0.68
	Puromycin	1.31	0.62
	Saquinavir	1.24	0.45
	Mean ± S.D.	1.17 ±0.21	0.59 ±0.20
GROUP B	Chloroquine	1.43	0.95
	Colchicine	1.30	0.86
	Domperidone	1.33	0.92
	Eletriptan	1.46	0.94
	GSK2	1.29	0.86
	GSK3	1.38	0.97
	Indacaterol	1.23	0.92
	Rh-123	1.30	0.82
	Salbutamol	1.47	0.88
	Salmeterol	1.60	0.80
	Mean ± S.D.	1.38^§^ ±0.11	0.89^¶^ ±0.06

Both Log K^IAM^ and MLV partitioning reflect molecular properties discriminating membrane interactions and, not surprisingly, significant negative correlations were observed with the calculated physico-chemical properties reflecting polarity. For example, the MLV partitioning possessing strong negative correlations with PSA (*P* = 0.005x10^−7^), H-Bond Total (*P* = 0.001x10^−6^), Abraham Basicity (*P* = 0.001x10^−4^) and H-Bond acceptor (*P* = 0.001x10^−2^).

### Intestinal Permeation of P-Gp Substrates: Impact of P-Gp Knockout

In parallel with the IPML absorption studies we examined the impact of *Mdr1a*/*1b* (−/−) knockout upon the intestinal permeation of our panel of compounds. We used an established Ussing chamber model comprising ileal segments isolated from the same mice as used in the IPML experiments.

Tissue viability of the intestinal segments was maintained over the 3 h permeation experiments with no intestinal segment displaying a drop in TEER greater than 10% from the initial pre-dosing readings. Across all preparations we found no significant difference (*P* > 0.05) in the TEER between the wild type (*Mdr1a*/*1b* +/+) and knockout (*Mdr1a*/*1b* −/−) tissue, i.e. respective TEER readings of 83 ± 21 Ω.cm^2^ and 79 ± 19 Ω.cm^2^_._ The lack of effect of P-gp deletion upon intestinal TEER is consistent with the findings of Stephens *et al*. (21) reporting no difference in the permeability of wild-type and *Mdr1a* (−/−) mouse intestinal segments to mannitol; *Mdr1a* being the more highly expressed Mdr1 product in the intestine.

**Fig. 4:**
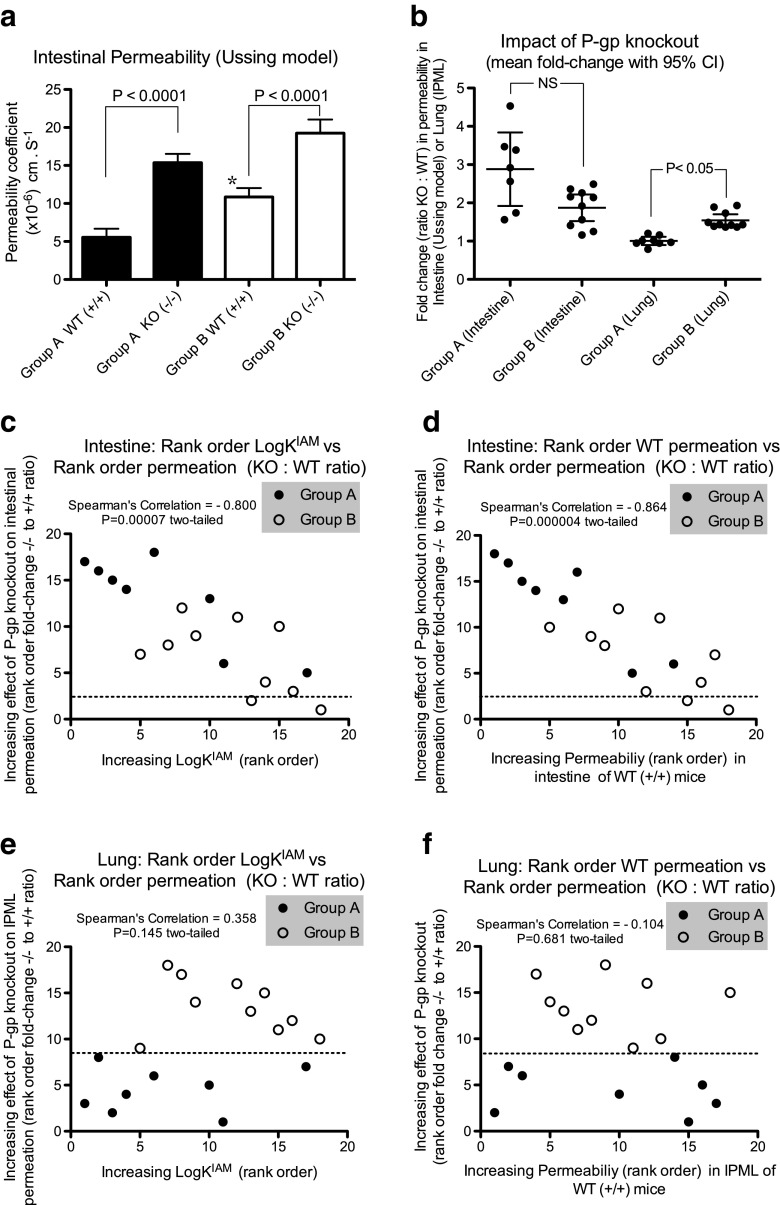
Comparative impact of *Mdr1a*/*1b* (−/−) knockout upon P-glycoprotein substrate permeation in intestinal ileal segments (Ussing chamber) and the IPML model. (**a**) Permeability of Group A compounds (filled bars) and Group B compounds (unfilled bars) across isolated mouse intestinal segments in WT (*Mdr1a*/*1b* +/+) and KO (*Mdr1a*/*1b* −/−) mice. Data represent mean ± S.D. with *n* = 8 for Group A compounds and *n* = 10 for Group B compounds. Data for each compound in any given *Mdr1a*/*1b* phenotype is derived from six to nine intestinal segments. Original data in Table III. *indicates statistical difference (*P* = 0.02) between the Group A WT and Group B WT; (**b**) Mean fold-change in permeation arising from P-gp knockout, expressed as KO: WT ratio. Mean value represented by horizontal line with associated 95% confidence interval. Also shown are the individual ratios for the Group A and B compounds for intestinal permeation (ratios of P_app_ values, *P* > 0.05) and for IPML absorption (ratios of AUC_0–30 min_ values, *P* < 0.05). Original data in Tables I (lung) and IV (intestine). For clarity the intestinal ratio data for the Group A compound saquinavir (10.07) has been excluded. Its inclusion would result in an increase in the mean ratio for the Group A (Intestine) data from 2.92 to 3.78 without changing the statistical inference; (**c** and **d**) Rank order correlations of the 18 P-gp substrates in the intestinal Ussing chamber model where on the y-axis is plotted the compound rank order of the increasing impact of P-gp knockout upon intestinal permeability. A greater rank indicates a greater impact of the KO phenotype on a compound’s permeation. On the x-axis is plotted: (**c**) the compound rank order for LogK^IAM^, where a greater rank indicates a greater LogK^IAM^. (**d**) the compound rank order for intestinal P_app_ in WT *Mdr1a*/*1b* (+/+) tissue, where a greater rank indicates a greater permeation. Plots derived from data in Tables III and IV, respectively; (**e** and **f**) Rank order correlations of the 18 P-gp substrates in the IPML model where on the y-axis is plotted the compound rank order of the increasing impact of P-gp knockout upon IPML absorption (AUC_0–30_). On the x-axis is plotted: (**e**) the compound rank order for LogK^IAM^; (**f**) the compound rank order for IPML absorption in WT *Mdr1a*/*1b* (+/+) tissue, where a greater rank indicates a greater absorption. Plots derived from data in Tables II and III. In plots 4c to 4f those compounds below the horizontal dashed line failed to show a significant (*P* > 0.05) change in permeation as a result of the KO, while those compounds above the line displayed a significant (*P* < 0.05) increase in permeation with the KO.

In contrast to the IPML data our studies with *Mdr1a*/*1b* (−/−) knockout in intestinal tissue resulted in significantly increased (*P* < 0.0001) permeabilities for both the Group A and Group B compounds (Fig. 4a, Table III), with an average 2.8-fold increase in permeability for the Group A compounds and a 1.9-fold increase for the Group B compounds (Fig. 4b). In keeping with the Group B compounds displaying a less polar character (consistent with faster transmembrane movement) these compounds showed a significantly greater (*P* = 0.02) permeability than the Group A compounds in the WT *Mdr1a*/*1b* (+/+) intestinal tissue, a difference that was however abolished with the *Mdr1a*/*1b* knockout (*P* > 0.05) (Fig. 4a). This suggests in the absence of P-gp a near comparable intestinal permeability between the Group A and B compounds, but in the presence of P-gp the intestinal permeability of the Group A compounds is more susceptible to P-gp-mediated efflux. The disparity in outcomes between the lung and intestinal models is exemplified in Fig. 4b where in the lung it is the absorption of the Group B compounds only that are affected (increased) by P-gp knockout, while in the intestine the permeability of both sets of compounds are increased, although here the Group A compounds appeared to be affected to a greater extent.

**Table III:** Permeability coefficients (P_app_) in the Ussing chamber model using intestinal segments isolated from either wild-type P-gp expressing (+/+) or P-gp knockout (−/−)mice. The 18 substrates are grouped into those compounds whose absorption in the IPML was unaffected by P-gp knockout (Group A) or those compounds whose absorption in the IPML was increased by P-gp knockout (Group B). Data represent the mean ± SD of *n* = 8 intestinal segments matched to the IPML experiments. Statistical analysis for intestinal P_app_ comparisons by unpaired T-test. Across all experiments there was no significant difference in the TEER values between the wild-type P-gp expressing (+/+) or P-gp knockout mice (−/−) with, respectively TEER = 83 ± 21 and 79 ± 19 Ω.cm^2^

		*Mdr1a*/*1b* (+/+) Permeability coefficient cm/s (x10^−6^)	*Mdr1a*/*1b* (−/−) Permeability coefficient cm/s (x10^−6^)	*P*-value	Ratio Permeability (−/−): (+/+)
GROUP A	Acrivastine	9.17 ± 2.87	14.32 ± 2.88	0.044	1.56
	Digoxin	6.04 ± 1.46	20.94 ± 2.94	0.0001	3.47
	Erythromycin	3.02 ± 1.15	13.70 ± 4.54	0.004	4.53
	GSK1	3.13 ± 1.25	10.57 ± 3.23	0.005	3.38
	Mitoxantrone	4.69 ± 1.06	13.70 ± 2.69	0.001	2.92
	Monensin	10.9 ± 1.78	19.06 ± 2.41	0.002	1.74
	Puromycin	5.83 ± 2.38	14.95 ± 2.33	0.002	2.56
	Saquinavir	1.56 ± 0.69	15.73 ± 3.52	0.0002	10.07
GROUP B	Chloroquine	14.95 ± 2.33	23.39 ± 4.18	0.012	1.56
	Colchicine	6.93 ± 3.15	14.95 ± 3.18	0.012	2.16
	Domperidone	10.78 ± 1.77	25.42 ± 8.04	0.012	2.36
	Eletriptan	4.90 ± 2.51	11.04 ± 2.17	0.010	2.26
	GSK2	8.18 ± 2.33	17.50 ± 2.91	0.002	2.14
	GSK3	12.2 ± 2.25	15.31 ± 2.25	0.102	1.25
	Indacaterol	15.2 ± 2.79	29.22 ± 4.40	0.002	1.92
	Rh-123	9.06 ± 1.81	22.55 ± 2.24	0.0001	2.49
	Salbutamol	10.6 ± 2.30	14.90 ± 2.16	0.034	1.41
	Salmeterol	15.8 ± 2.16	18.33 ± 2.38	0.170	1.16

Figure 4c shows for the 18 compound panel the rank order relationship between LogK^IAM^ (x-axis) and the impact of P-gp knockout upon intestinal permeability (y-axis). A strong negative correlation (Spearman’s Coefficient − 0.800, *P* = 0.00004) was observed with the inference that compounds with a lower membrane affinity are more susceptible to P-gp-mediated efflux in the intestine, and vice versa; substituting LogK^IAM^ with the MLV partitioning parameter produced a similar trend. When LogK^IAM^ is substituted for the rank order of compound intestinal permeation in the WT *Mdr1a*/*1b* (+/+) mice (x-axis) a similar strong negative correlation (Spearman’s Coefficient − 0.864, *P* = 0.000004) is obtained (Fig. 4d). Both Fig. 4c and d show for the intestinal tissue the tendency for the more polar Group A substrates to be affected to a greater extent by P-gp.

It should be noted that while all Group A and B compounds showed a consistent trend for increased permeation across the intestinal tissue of the knockout mice the data for two of the Group B compounds, GSK3 and salmeterol, failed to reach statistical significance (*P* > 0.05) (Table III). These compounds are represented in Fig. 4c and d as those falling below the horizontal dashed line. The reason for this is unclear although not inconsistent with accepted understanding, i.e., GSK3 possesses one of the most non-polar physico-chemical profiles within the panel as a whole, and together, GSK3 and salmeterol display the greatest membrane interactions in one or other of this study’s membrane affinity/MLV partitioning experiments (Table II).

Figure 4e and f show comparative correlation plots for the IPML experiments. Here no relationship was seen between the rank order LogK^IAM^ and the impact upon IPML permeability arising from P-gp knockout (Fig. 4e); substituting LogK^IAM^ with the MLV partitioning produced a similar trend. A lack of correlation was also evident where the LogK^IAM^ data (x-axis) was replaced by the rank order for compound permeation in the WT *Mdr1a*/*1b* (+/+) lungs (Fig. 4f). Figure 4e and f highlight again that it is only the Group B compounds, i.e. those displaying a less polar character, whose permeation in the lung is affected by P-gp.

### Lung Retention of P-Gp Substrates

**Fig. 5:**
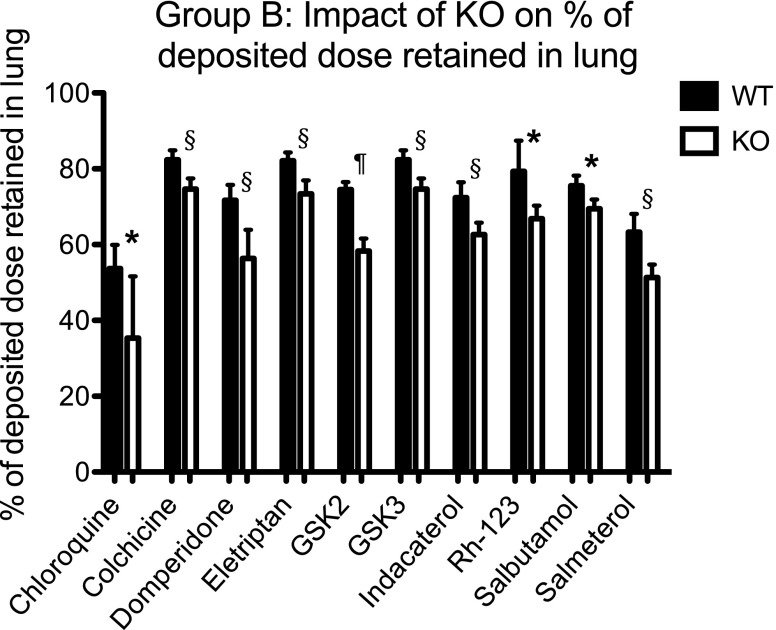
Lung retention of Group B compounds. The % of lung deposited dose retained in the IPML lungs of WT *Mdr1a*/*1b* (+/+) and KO *Mdr1a*/*1b* (−/−) mice at the end of the 30 min IPML absorption experiments. Data represent mean ± S.D., *n* = 4 to 6 mice for each treatment arm. * indicates statistical difference at *P* < 0.05, ^§^ indicates statistical difference at *P* < 0.01, ^¶^ indicates statistical difference at *P* < 0.001.

Figure 5 shows for the Group B compounds the % of deposited dose associated with the lung tissues at the end of the IPML experiments; the data was determined by mass balance calculations accounting for drug remaining in the dosing syringe and tracheal cannula, and the mass of compound absorbed to the perfusate. The lung retention data highlights significant (*P* < 0.05) differences for each of the Group B compounds between *Mdr1a/1b* (+/+) WT and *Mdr1a/1b* (−/−) KO mice. While such differences in lung retention between the P-gp phenotypes are significant across the Group B compounds as a whole they account for modest changes. For example, with respect to those inhaled molecules within our panel the absolute difference between the % of deposited dose retained in the WT *versus* the KO lungs ranged from 16% for GSK2 (i.e. 74.5% - 58.3%) to 6% for salbutamol (i.e. 75.5% - 69.5%). However even comparatively small differences reported in the context of the current experimental design may become more noteworthy in a clinical setting.

### Development of a QSAR Model to Predict Differential Absorption of P-Gp Substrates in the IPML

To identify the principal physico-chemical determinant(s) underpinning the functional differentiation between the Group A and Group B P-gp substrates, and to build a predictive model based on structure alone we undertook multivariate analysis by constructing an orthogonal PLS Discriminant Analysis (oPLS-DA) model comprising the 13 physico-chemical properties for all 18 compounds (Table I).

**Fig. 6:**
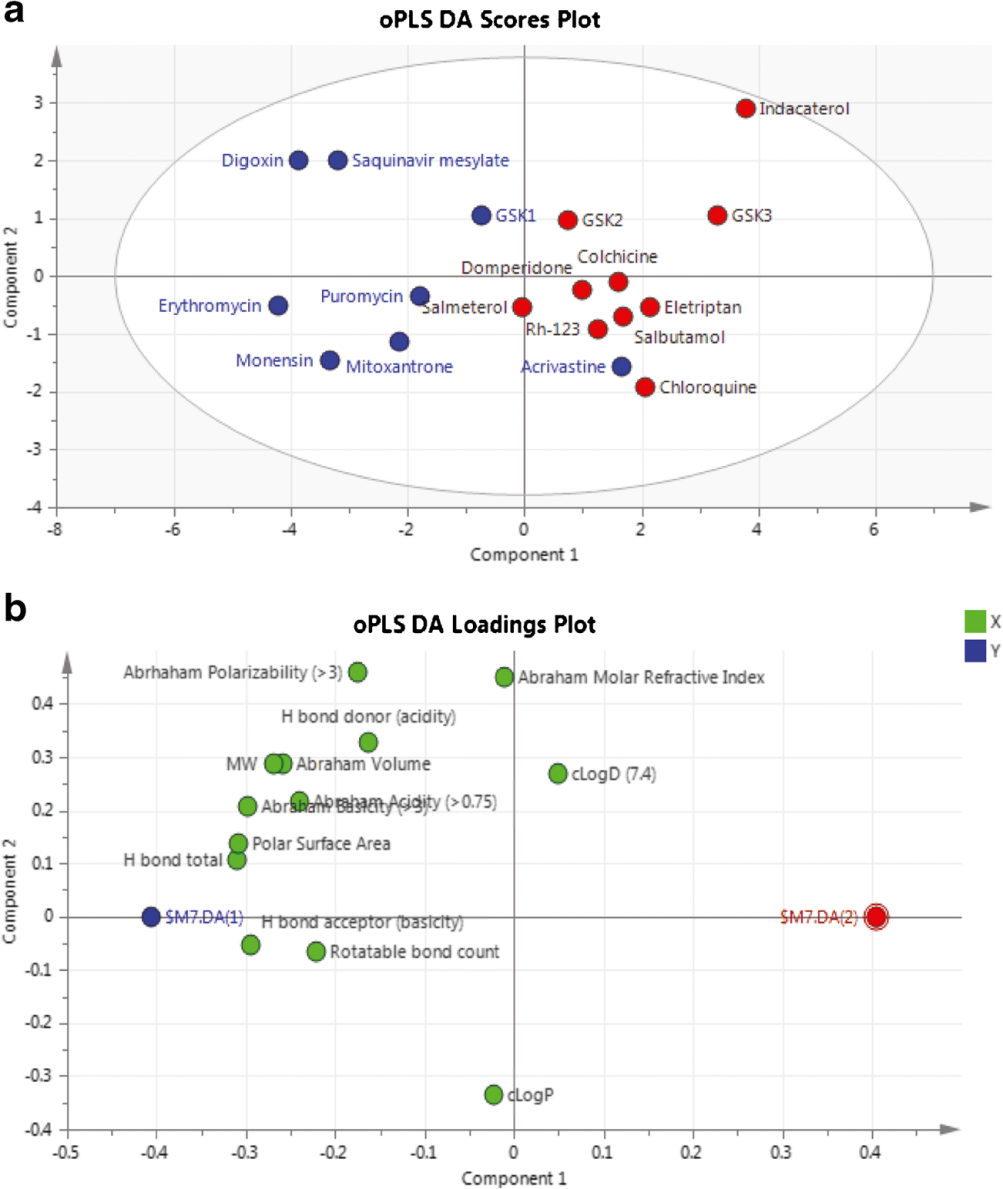
QSAR Modeling using orthogonal PLS Discriminant Analysis (oPLS-DA). (**a**): The Scores plot from the resulting oPLS model generated within SIMCA-P+ on the 18 P-gp substrates in our panel showing differentiation between the Group A and Group B compounds based on their computed physico-chemical properties. Group A compounds represented by the blue symbol, Group B by the red symbol. (**b**): The Loadings plot from the resulting oPLS model highlights the contribution of each of the physico-chemical descriptors to the model components, with the increasing magnitude in the values of the descriptors towards the left of the plot associated with Group A characteristics.

We identified a comprehensive series of physico-chemical descriptors that correlate with whether the compounds show a differentiation in their pulmonary absorption in the IPML model between *Mdr1a/1b* (+/+) WT and *Mdr1a/1b* (−/−) KO mice. The fitted oPLS model (R2 = 059, Q2 = 0.53 *n* = 18) provided excellent separation between Group A and Group B substrates with only one outlier, the Group A compound acrivastine. The Scores Plot of the resulting oPLS model (Fig. 6a) displays the relationships between the different compounds; with Group A compounds represented by Blue symbols and Group B compounds by Red symbols. Here acrivastine, lying on the right hand side of the plot is shown to be more closely related to the Group B compounds based on the 13 physico-chemical descriptors (x-axis variables) used to build the model, yet it is an outlier because it behaves as a Group A compound in the IPML with its absorption unaffected by P-gp knockout.

The explanation as to what accounts for the separation between the 18 compounds on the Scores Plot is highlighted in the Loadings Plot (Fig. 6b). Again the attributed variability is described by component 1 along the x-axis and therefore an increasing magnitude in the values of the descriptors towards the left of the plot are associated with characteristics of the Group A compounds. Those descriptors positively correlated with Group A characteristics are those associated with increased polarity, i.e. P-gp substrates more likely to display lower membrane affinity and reduced transmembrane permeation rates, the very characteristics that would be indicative of P-gp limiting substrate permeation in the intestine.

The statistical output from the oPLS model displayed a R^2^ of 0.59 and a Q^2^ (i.e. predictivity) of 0.53. Considering the low number of observations upon which the model was built (*n* = 18 compounds) the statistics are sufficient for a semi-quantitative ranking and classification-based predictive assessment.

The oPLS model was then used to predict whether 129 known P-gp substrates were likely to display characteristics more like the Group A or Group B compounds. The 129 compounds, their physico-chemical properties and the resulting oPLS model predictions are shown in full in Supplementary Table S6. The vast majority of compounds (101 out of 129) are predicted to be Group B like, i.e. substrates that if administered into the lung would display pulmonary absorption likely to be affected by P-gp. Within the panel of 129 molecules was the P-gp substrate, loperamide, which was forecast to behave as a Group B compound. Loperamide was not part of the current 18 member panel of substrates but in earlier independent IPRL investigations its pulmonary absorption was shown to be affected by P-gp (16).

## Discussion

Using lungs derived from *Mdr1a*/*1b* genetic knockout mice we explored the impact of pulmonary P-gp upon the absorption of an 18 member panel of P-gp substrates administered into the airways of an isolated perfused mouse lung (IPML). Solely on the basis of the impact of P-gp upon a substrate’s absorption within the IPML we defined the panel members as either ‘Group A’ compounds i.e. those whose pulmonary absorption was unaffected by Mdr1a/1b knockout, or ‘Group B’ compounds, i.e. those whose pulmonary absorption was limited by P-gp, specifically absorption was increased by Mdr1a/1b knockout. We explored if the physico-chemical properties of these two groups of compounds were distinguishable. We found the Group A compounds to possess a distinctly more polar character and significantly lower phospholipid membrane affinities and transmembrane movement rates compared to the Group B compounds.

To address if the Group A and B compounds could be differentiated on the basis of intrinsic P-gp binding kinetics we used artificial membrane models constituted by human (MDR1) and mouse (both *Mdr1a* and *Mdr1b*) P-gp. Although the quantitative nature of such in-vitro kinetic parameters may not be directly translatable to the in-vivo barrier, the results substantiated the similar and overlapping mouse *Mdr1a* and *Mdr1b* (and indeed human MDR1) binding interactions between the Group A and Group B compounds. We also examined the interactions of the panel of P-gp substrates with mouse *Bcrp*. Human (BCRP) and rodent (*Bcrp*) lungs have been shown to express this transporter (3), with at least in humans BCRP expression seen in alveolar pneumocytes (26) but with little to no expression in bronchial epithelium (9,26). We found only four of the 18 compounds (two from each of Group A and Group B) to show evidence of *Bcrp* interactions, the very four compounds where some literature evidence exists that supports such interactions either in the context of a substrate or an inhibitor (27–30); the most recognised BCRP/Bcrp substrate within our panel being mitoxantrone. Importantly, in respect to *Bcrp* interactions there was no selective bias towards the Group A compounds, and as such we consider potential *Bcrp* efflux in the lung per se was not a determining factor responsible for the lack of effect of *Mdr1a*/*1b* deletion to result in increased pulmonary absorption for the eight Group A compounds.

We next questioned how the differing physico-chemical properties of the Group A and Group B compounds may determine susceptibility to P-gp efflux in the lung compared to the intestine. For the latter we used an Ussing chamber model with isolated intestinal (ileal) segments derived from the *Mdr1a*/*1b* knockout mice. We found the relationship between substrate physico-chemical properties and absorption in the lung to be dissimilar to that evident in the intestine. Namely, in the intestine it was the permeation of the more polar P-gp substrates (Group A compounds) that was more susceptible to the effects of P-gp efflux, a finding consistent with previously defined relationships established in various intestinal preparations (17,31,32). In contrast, in the lung the absorption of the least polar substrates (Group B compounds) was most affected by P-gp with the pulmonary absorption of the Group A substrates remaining unaffected.

We suggest it is the interplay between the substrates’ differential physico-chemical properties coupled with the differing nature of the absorption barriers which underpins the discordant findings we report above: The initial step in the passive transcellular permeation of a compound is its interaction with the outer membrane leaflet. The more non-polar a compound the greater its affinity for membrane binding which will increase the concentration of the compound in the outer membrane leaflet; the donor ‘reservoir’ for the compound’s transmembrane movement. As such increases in a compound’s membrane affinity, while not the rate-limiting step, may be predicted to increase a compound’s transmembrane permeability rate (33,34). At least in the intestine it is apparent that the effect of P-gp upon the passive transcellular permeation of a substrate is influenced by the opposing considerations of the intrinsic efficiency of P-gp to efflux the substrate and the substrate’s passive transmembrane permeability rate (17,31,32). Specifically, an increased passive transmembrane permeability rate is associated with a reduced membrane dwell time and a reduced net effect of P-gp upon a substrate’s overall permeation. This same relationship also appears operative for membrane transport within induced MDR cell phenotypes (20,35). It also extends to subsets of P-gp substrates whose transmembrane dwell time is so short that intestinal transcellular permeation appears to be essentially uninfluenced by P-gp (36). The above understanding is consistent with the intestinal data we report here. Indeed, our overall findings do not challenge the above principle when this manifestly applies to the process of transmembrane transport itself. There are however some notable differences between the intestinal and lung barriers that are relevant to the interpretation of our data.

There is a general recognition that polar molecules administered into the lung airways show a greater permeation across lung epithelium than across other epithelial barriers, a finding considered to reflect a greater passive permeation through paracellular pathways. This is substantiated by kinetic evidence from a range of species, for an assortment of molecule classes and by a number of different laboratories, for example (13,37,38) and reviewed in (39–42). One such example is the pulmonary absorption of mannitol and inulin, both absorbed from the lung at a faster rate and to a greater extent (50% and 17% of administered dose, respectively) than from the small intestine (<2%) (38). Another example is from the work of Tronde *et al*. (13) reporting that compounds having low oral permeability in the Caco-2 cell model (Papp values <1 x 10^−6^ cm/s), such as terbutaline, cromolyn and cyanocobalamin, show high permeability across the lung. Indeed, there are a number examples of polar compounds possessing a PSA ≥120 Å^2^ and recognised to display <10% oral absorption but which nevertheless display >80% pulmonary bioavailability.

**Fig. 7:**
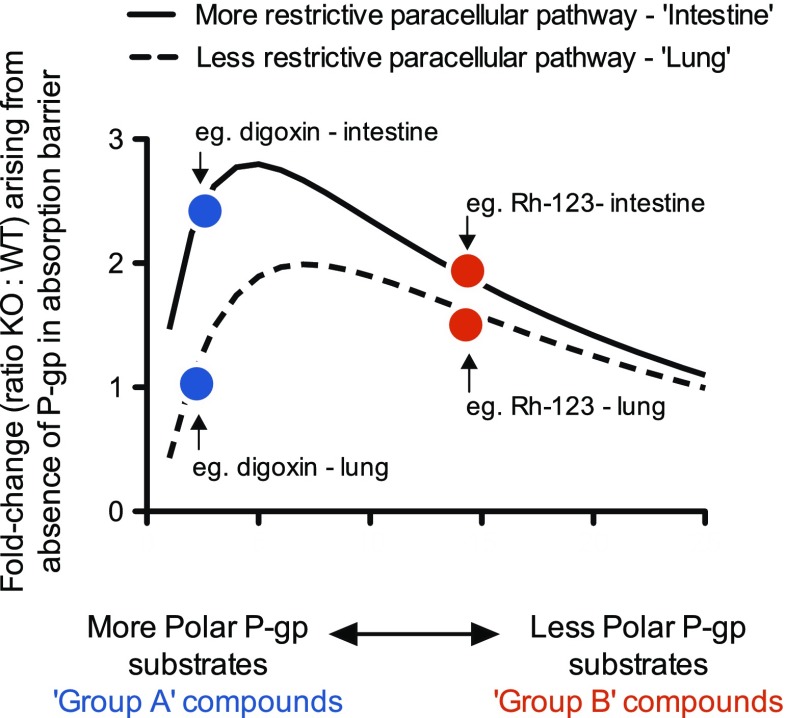
Putative model of the combined impact of epithelial barrier paracellular permeability and compound physico-chemical properties upon the absorption of P-gp substrates: For a more restrictive paracellular pathway (e.g. intestine, Solid line) P-gp efflux will have a greater net effect upon overall barrier absorption for the more polar substrate(s) (Blue symbol - e.g. digoxin) compared to the less polar substrate(s) (Red symbol - e.g. Rh-123). Alternatively, for the barrier with a less restrictive ‘leakier’ paracellular pathway (e.g. lung, Dashed line) P-gp efflux will have a greater net effect upon overall barrier absorption for the less polar substrate(s) (Red symbol - e.g. Rh-123) compared to the more polar substrate(s) (Blue symbol - e.g. Digoxin). A fold-change of 1 (y-axis) is indicative of no net effect upon overall absorption arising from the deletion of P-gp, i.e. P-gp has no impact upon a substrate’s overall absorption.

A tentative model we therefore propose to explain our results is represented in Fig. 7. Specifically, it considers the following: a) less polar P-gp substrates will be predominantly absorbed across a barrier by the passive transcellular route and as such be exposed to plasma membrane P-gp; b) some of these substrates will display a greater membrane affinity, a higher transmembrane permeation rate and as such experience a lesser net effect of P-gp efflux, and vice versa; c) with increasing substrate polarity the net effect of P-gp efflux will be observed to increase as the substrate’s membrane dwell time is extended; d) as polarity increases further, however, then more time will be required for the substrate to achieve high enough concentrations in the membrane for the net effect of P-gp efflux to be measured. Importantly a kinetic consequence of this will be a greater proportion of the polar substrate available for passive absorption via the paracellular pathway avoiding exposure to P-gp.

Applying the above to our data (Fig. 7) we may view the intestine as a barrier with a more restrictive paracellular pathway. Consequently, in the intestine there is reduced paracellular transfer for the more polar P-gp substrates. As such the net effect of P-gp upon overall intestinal absorption will be seen to be greater for the more polar substrates (e.g. Group A compounds such as digoxin) compared to the less polar substrates (e.g. Group B compounds such as Rh-123). For a barrier with an apparently less restrictive ‘leakier’ paracellular pathway (e.g. lung) a greater proportion of the more polar P-gp substrates will be absorbed by the paracellular route and avoid plasma membrane P-gp efflux. As such the net effect of P-gp upon overall lung absorption will be seen to be greater for the less polar substrates (i.e. Group B). It is only when the same set of P-gp substrates are studied across different absorption barriers will any such distinct barrier-dependent physico-chemical requirements be observed.

The question of how the relative expression levels of P-gp between lung and intestinal barriers may impact our interpretation warrants some comment. It is fair to conclude from transcriptional and protein expression data (3–9) as well in-vitro transport studies (reviewed in (1)), that functional P-gp expression in lung epithelium will likely be significantly less than in ileal enterocytes. However, a lower P-gp expression in the lung barrier would not in its own right be the basis for the differential and transposed physico-chemical relationships we have observed in this current work. Indeed, regardless of the expression level of P-gp it would remain true that the more polar substrates if accessing the plasma membrane would be those that remain most affected by P-gp. Although a reduced P-gp level in any barrier would make less distinct the impact of P-gp between polar and less polar substrates. In summary, against a background of divergent paracellular properties between barriers the different levels of P-gp expression may magnify to a lesser or greater extent the overall impact of P-gp efflux upon barrier absorption, although the trend as outlined in the scheme of Fig. 7 would remain generally similar.

By applying multivariate analysis to the panel of 18 P-gp substrates we identified a comprehensive series of physico-chemical descriptors that correlate with whether the compounds show a differentiation in their pulmonary absorption in the IPML model between the *Mdr1a/1b* (+/+) WT and *Mdr1a/1b* (−/−) KO mice. The main attributes of our multivariate approach, oPLS and its extension to oPLS-DA, have been described elsewhere (43), with the attraction of this method being its suitability for classification of data that have multi-collinear and noisy variables. In particular the oPLS-DA algorithm will model the discriminatory components and the Y-orthogonal components separately, which makes interpretation of the model straightforward as all the information relating to the classification will be contained along the x-axis of the Loadings plot. The oPLS model we generated was used to predict whether 129 known P-gp substrates if administered into lung airways would likely display characteristics more like the Group A or the Group B compounds. The vast majority of compounds (101 out of 129) are predicted to be Group B like, i.e. substrates if administered into the lung would display pulmonary absorption that would be affected by P-gp. These oPLS model predictions included eight P-gp substrates currently licenced as inhaled medicines: the beta-adrenoreceptor agonists salbutamol, salmeterol, indacaterol, and the glucocorticoids beclomethasone, budesonide, ciclesonide, fluticasone and mometasone. All eight were identified as fitting the profile for Group B compounds. This current QSAR model in combination with other QSAR models in this area (44) may collectively predict from molecular structure alone both the extent of pulmonary absorption and the impact of pulmonary P-gp. As such these models advance our understanding of the impact of P-gp in the airways relevant to profiling of inhaled drug discovery candidates.

For inhaled locally acting drugs it is the impact of an efflux mechanism upon intra-luminal pulmonary disposition and unbound concentrations in local tissue compartments and epithelial lining fluid (ELF) that will be of relevance in local PK-PD relationships (1,45). The complexities regulating intra-pulmonary kinetics, exemplified by inhaled corticosteroids, are the subject of notable commentaries by Derendorf and Hochaus (46–48). In the current work we determined P-gp to increase the lung retention for some inhaled drugs/drug candidates by up to 16% of the deposited dose. However, even such comparatively small increases in the context of the current short (30 min) experimental design may become more noteworthy in a clinical setting. For example, the impact of P-gp-mediated efflux upon substrate intra-luminal concentrations will become progressively more significant as local concentrations decrease at later time points following inhaled administration. Indeed the dosage indications for most of the inhaled glucocorticoids are suggestive that initial concentrations in human ELF maybe sufficiently high so as to partially overcome a potential P-gp efflux capacity.

In this context we rule out any potential saturation of P-gp within the IPML as the basis for the lack of effect of P-gp upon the pulmonary absorption of the Group A substrates. Simply, all non-radiolabelled compounds (be they Group A or B compounds) were administered at equivalent doses estimated to achieve an initial indicative concentration in the IPML ELF approximating 25 μM. These same concentrations were used in the intestinal Ussing chamber studies. We also found no distinction in the *Mdr1a*/*Mdr1b* binding kinetics between the two groups. Further, the archetype Group A compound, digoxin, was delivered at a radiolabel dose of 16 pmoles, equating to an estimated initial concentration in ELF approximating 0.3 μM. Neither should respiratory mucus be viewed as the basis of the differential outcomes we found. Mucus comprises amongst other elements a high proportion of water and mucin glycoprotein polymer, the fibres of which form a fibrillar network with inter-fibre water-filled pores in the 100 nm range (49). While mucus represents a permeability barrier to macromolecules or supramolecular entities, through physical size restriction and hydrophobic and ionic interactions, small molecules such as those in the current study are less prone to suffer diffusional hindrance within the mucus barrier.

We undertook a pilot in-vivo experiment dosing to wild-type (*Mdr1a*/*1b* +/+) and knockout (*Mdr1a*/*1b* −/−) mice the archetype compounds, digoxin and Rh-123, via intra-nasal instillation; a route delivering a significant fraction of dose to the respiratory tract (see Supplementary Pilot In-vivo experiment). Consistent with the IPML study, and in agreement with Shanker’s digoxin lung clearance data (50), the clearance of digoxin from the in-vivo mouse lungs was rapid with digoxin lung levels similar between the wild-type and knockout mice at 1 h post-instillation, but undetectable thereafter. Indeed, it is the lung clearance profiles that may be more reliable and informative following the intra-nasal instillation dosing. For Rh-123 we measured lung levels up to 12 h post-instillation. The bi-exponential Rh-123 lung clearance curves displayed a t^1^/_2_ for the initial decline (over the first 3 h) that was slower (t^1^/_2_ 136 min) in WT animals than in KO animals (t^1^/_2_ 107 min), thereafter the terminal decline phase was similar but more prolonged (t^1^/_2_ 500 min) in both the WT and KO animals. Cationic lipophilic drugs such as Rh-123 tend to accumulate in the lungs to form slowly eluting pools (51,52). One interpretation of our pilot data is that the initial more rapid decline phase represents loss of Rh-123 from the airways across lung epithelium to blood, a process influenced by P-gp in the airway epithelium. The slower terminal phase represents loss of Rh-123 from slowly eluting pools in the lung parenchyma, with Rh-123 having previously passed across the airway epithelium this clearance phase is not subject to P-gp effects. The pilot study shows some encouragement for conducting a fuller in-vivo investigation particularly when aligned to studying the potential impact of pulmonary P-gp efflux upon efficacy and duration of action.

This current study has focussed on P-gp efflux from the lung epithelium to airway lumen, however Roerig *et al*. (53) proposed P-gp to serve as a barrier limiting uptake of the P-gp substrate rhodamine 6G into the lung from blood. In our own preparative IPRL and IPML experiments we found no evidence of P-gp mediated efflux limiting uptake to the lung from the perfusate for either Group A or Group B compounds. This is consistent with in-vivo whole body disposition studies reported in *Mdr1* knockout mice (54–57) where, following i.v. or oral dosing routes, the lung to blood distribution ratios for those P-gp substrates so far studied indicate *Mdr1* knockout to have no significant impact upon lung accumulation. Nevertheless, this area warrants further study particularly given the potential role that lung P-gp may have in modulating drug-induced toxicities arising from systemic administration.

## Conclusion

We have demonstrated that pulmonary P-gp is functional in an intact lung impeding the absorption and increasing the lung retention of a subset of P-gp-substrates administered in the airways. Multivariate analysis identified a comprehensive series of physico-chemical descriptors that correlate with whether the absorption of a substrate will be affected by P-gp or not. The contribution of pulmonary P-gp to the lung absorption of airway administered substrates appears to be defined by a distinctly different physico-chemical relationship than that observed in the intestine. The QSAR model built upon the current dataset was used to predict the likelihood of 129 other known P-gp substrates undergoing P-gp-dependent pulmonary disposition. Within this set were included marketed pulmonary delivered drugs, of which there were eight and all of which were predicted to display P-gp mediated modulation in their pulmonary absorption. The research advances our understanding of the impact of P-gp in the airways relevant to profiling of inhaled drug discovery candidates. The findings also highlight a potential for P-gp mediated pulmonary disposition in the clinic.

## Electronic Supplementary Material

Supplementary Figure S1 **Genotype and phenotype of mdra1/b (−/−) mice:** Fig. 1 A shows agarose gel electrophoresis of all possible genotypes of the FVB/B6 hybrids using primers described in Table I. Lanes 1 and 5 are loaded with the 1 kb + ladder. Lanes 2–4 are for the *Mdr1a* gene and show: Lane 2 - homozygous wild type with product at 269, Lane 3 - heterozygote with products at 269 and 461, Lane 4 - homozygous knockout with product at 461 only. Lanes 6–8 are for the *Mdr1b* gene and show: Lane 6 - homozygous wild type with product at 540, Lane 7 - heterozygote with products at 540 and 453, Lane 8 - homozygous knockout with product at 453 only. Fig. 1 B shows pulmonary absorption of [^14^C]-mannitol from the airways of the isolated perfused mouse lung (IPML) model in CD1 mice, wild type FVB/B6 mice and *Mdr1a*/*1b*(−/−) knockout FVB/B6 mice. Data represent mean ± S.D., *n* = 6 for CD1 mice, and *n* = 12 for both sets of FVB/B6 mice. (GIF 571 kb)

High Resolution Image (EPS 1337 kb)

Supplementary Figure S2 **Pulmonary absorption profiles in the IPML model of Group B compounds**. (2A) Chloroquine, (2B) Domperidone, (2C) Colchicine, (2D) Eletriptan, (2E) Rh-123, (2F) Indacaterol. Data are mean ± S.D., *n* = 4–6. * indicates *P* < 0.05. The lines indicate the non-linear model fits to the observed data. Solid line (closed symbols) for the *Mdr1a*/*1b* (+/+) data. Dashed line (open symbols) for the *Mdr1a*/*1b* (−/−) data. (GIF 155 kb)

High Resolution Image (EPS 236 kb)

Supplementary Figure S3 **Pulmonary absorption profiles in the IPML model of Group B compounds.** (3A) Salbutamol, (3B) Salmeterol, (3C) GSK2, (3D) GSK3. Data are mean ± S.D., *n* = 4–6. * indicates *P* < 0.05. The lines indicate the non-linear model fits to the observed data. Solid line (closed symbols) for the *Mdr1a*/*1b* (+/+) data. Dashed line (open symbols) for the *Mdr1a*/*1b* (−/−) data. (GIF 95 kb)

High Resolution Image (EPS 184 kb)

Supplementary Figure S4 **Pulmonary absorption profiles in the IPML model of Group A compounds.** (4A) Monensin, (4B) Saquinavir, (4C) Mitoxantrone, (4D) Puromycin. Data are mean ± S.D., *n* = 4–6. * indicates *P* < 0.05. The lines indicate the non-linear model fits to the observed data. Solid line (closed symbols) for the *Mdr1a*/*1b* (+/+) data. Dashed line (open symbols) for the *Mdr1a*/*1b* (−/−) data. (GIF 102 kb)

High Resolution Image (EPS 182 kb)

Supplementary Figure S5 **Pulmonary absorption profiles in the IPML model of Group A compounds.** (5A) [^3^H] digoxin, (5B) Erythromycin, (5C) GSK1, (5D) Acrivastine. Data are mean ± S.D., *n* = 4–6. * indicates *P* < 0.05. The lines indicate the non-linear model fits to the observed data. Solid line (closed symbols) for the *Mdr1a*/*1b* (+/+) data. Dashed line (open symbols) for the *Mdr1a*/*1b* (−/−) data. (GIF 95 kb)

High Resolution Image (EPS 184 kb)

Supplementary Table S1 (DOCX 16 kb)

Supplementary Table S2 (DOCX 18 kb)

Supplementary Table S3 (DOCX 19 kb)

Supplementary Table S4 (DOCX 21 kb)

Supplementary Table S5 (DOCX 23 kb)

Supplementary Table S6 (EPS 630 kb)

ESM 1 (DOCX 105 kb)

## ABBREVIATIONS

ACN: Acetonitrile
BCRP,*Bcrp*: Breast cancer resistance protein
BSA: Bovine serum albumin
ELF: Epithelial lining fluid
F: Extent of bioavailability
GSK: GlaxoSmithKline
IAM: Immobilised artificial membrane
IPL: Isolated perfused lung
IPML: Isolated perfused mouse lung
IPRL: Isolated perfused rat lung
i.p.: Intra-peritoneal
Ka: First-order aborption rate constant
KO: Knockout
LC-MS/MS: Liquid chromatography mass spectrometry/tandem mass spectrometry
LLD: Lower limit of detection
LLQ: Lower limit of quantitation
MDR, *Mdr*: Multidrug resistant
MeOH: Methanol
MLV: Multi-lamellar vesicle
oPLS-DA: Orthogonal Projections to Latent Structures Discriminant Analysis
PCR: Polymerase chain reaction
PK-PD: Pharmacokinetics - Pharmacodynamics
P-gp: P-glycoprotein
PSA: Polar surface area
QSAR: Quantitative structure activity relationship
TEER: Transepithelial electrical resistance
WT: Wild type
