# Supplementary figures and images for: The Differential Absorption of a Series of P-Glycoprotein Substrates in Isolated Perfused Lungs from Mdr1a/1b Genetic Knockout Mice can be Attributed to Distinct Physico-Chemical Properties: an Insight into Predicting Transporter-Mediated, Pulmonary Specific Disposition
Source: Pharm Res. 2017 Jul 12;34(12):2498–516. doi: 10.1007/s11095-017-2220-5 (PMC5736782; doi:10.1007/s11095-017-2220-5)

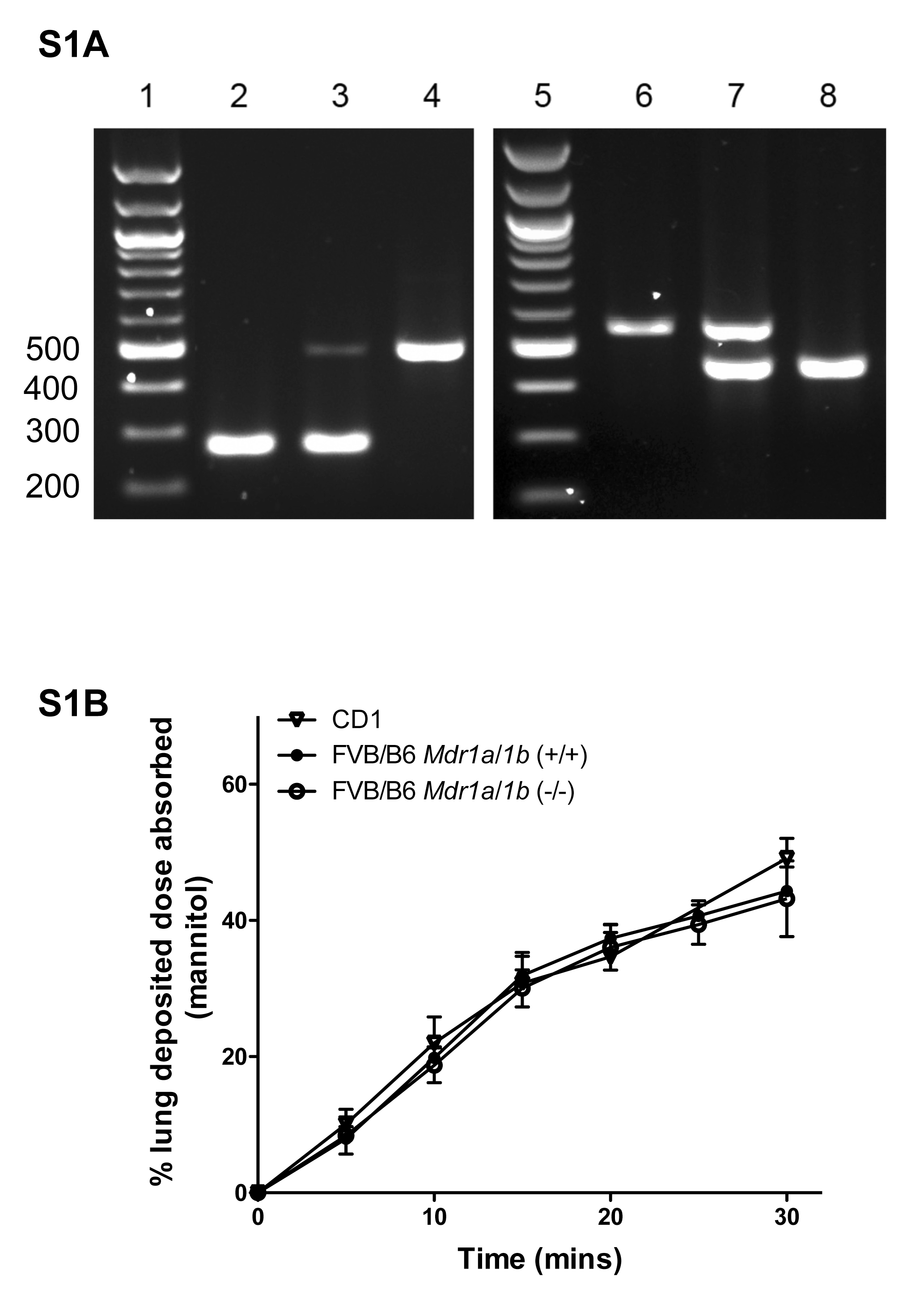

Supplement: Supplementary file 1 — Genotype and phenotype of mdra1/b (−/−) mice: Fig. 1 A shows agarose gel electrophoresis of all possible genotypes of the FVB/B6 hybrids using primers described in Table I. Lanes 1 and 5 are loaded with the 1 kb + ladder. Lanes 2–4 are for the Mdr1a gene and show: Lane 2 - homozygous wild type with product at 269, Lane 3 - heterozygote with products at 269 and 461, Lane 4 - homozygous knockout with product at 461 only. Lanes 6–8 are for the Mdr1b gene and show: Lane 6 - homozygous wild type with product at 540, Lane 7 - heterozygote with products at 540 and 453, Lane 8 - homozygous knockout with product at 453 only. Fig. 1 B shows pulmonary absorption of [14C]-mannitol from the airways of the isolated perfused mouse lung (IPML) model in CD1 mice, wild type FVB/B6 mice and Mdr1a/1b(−/−) knockout FVB/B6 mice. Data represent mean ± S.D., n = 6 for CD1 mice, and n = 12 for both sets of FVB/B6 mice. (GIF 571 kb) [file 11095_2017_2220_Fig8_ESM.gif]

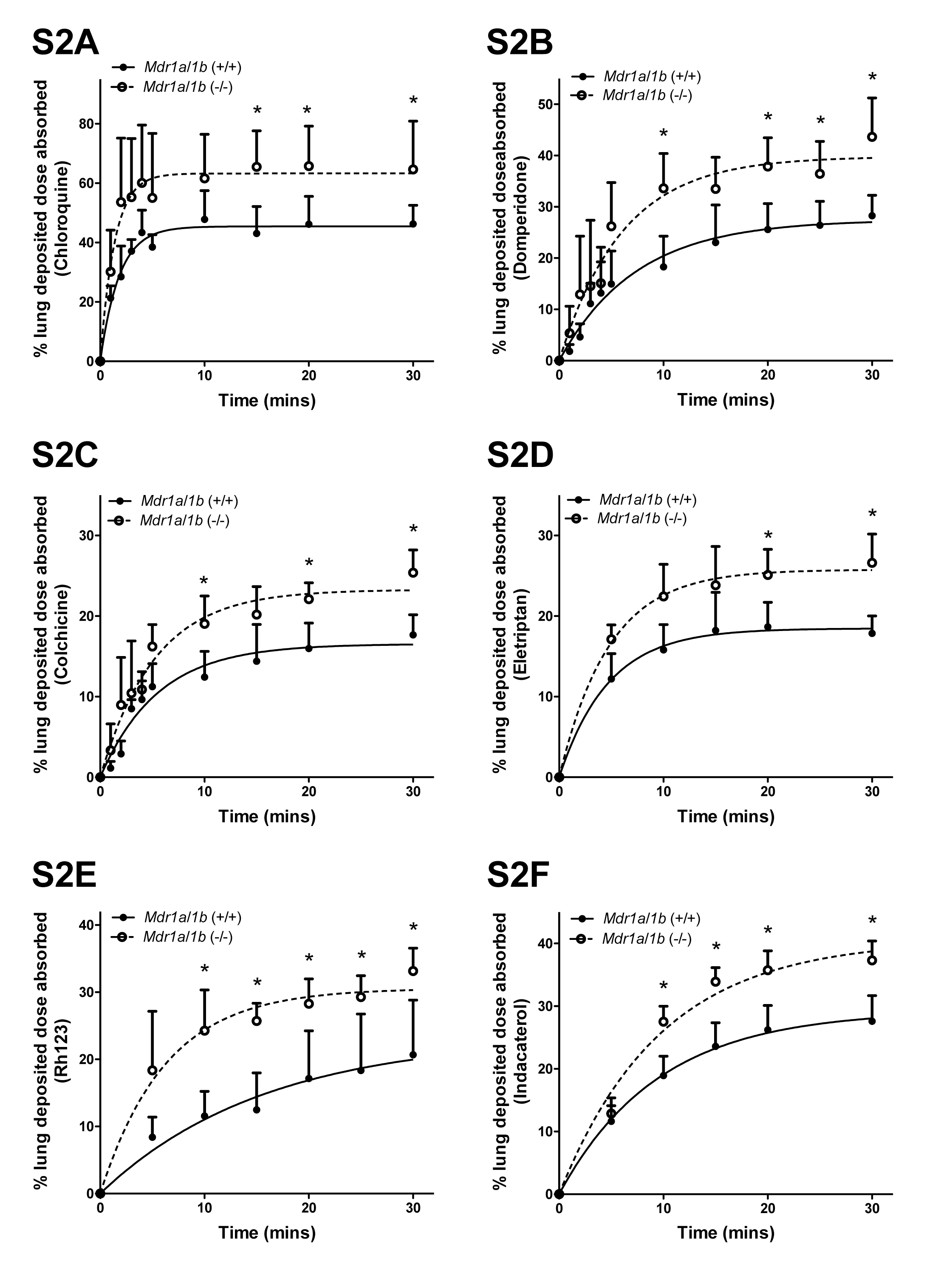

Supplement: Supplementary file 3 — Pulmonary absorption profiles in the IPML model of Group B compounds. (2A) Chloroquine, (2B) Domperidone, (2C) Colchicine, (2D) Eletriptan, (2E) Rh-123, (2F) Indacaterol. Data are mean ± S.D., n = 4–6. * indicates P < 0.05. The lines indicate the non-linear model fits to the observed data. Solid line (closed symbols) for the Mdr1a/1b (+/+) data. Dashed line (open symbols) for the Mdr1a/1b (−/−) data. (GIF 155 kb) [file 11095_2017_2220_Fig9_ESM.gif]

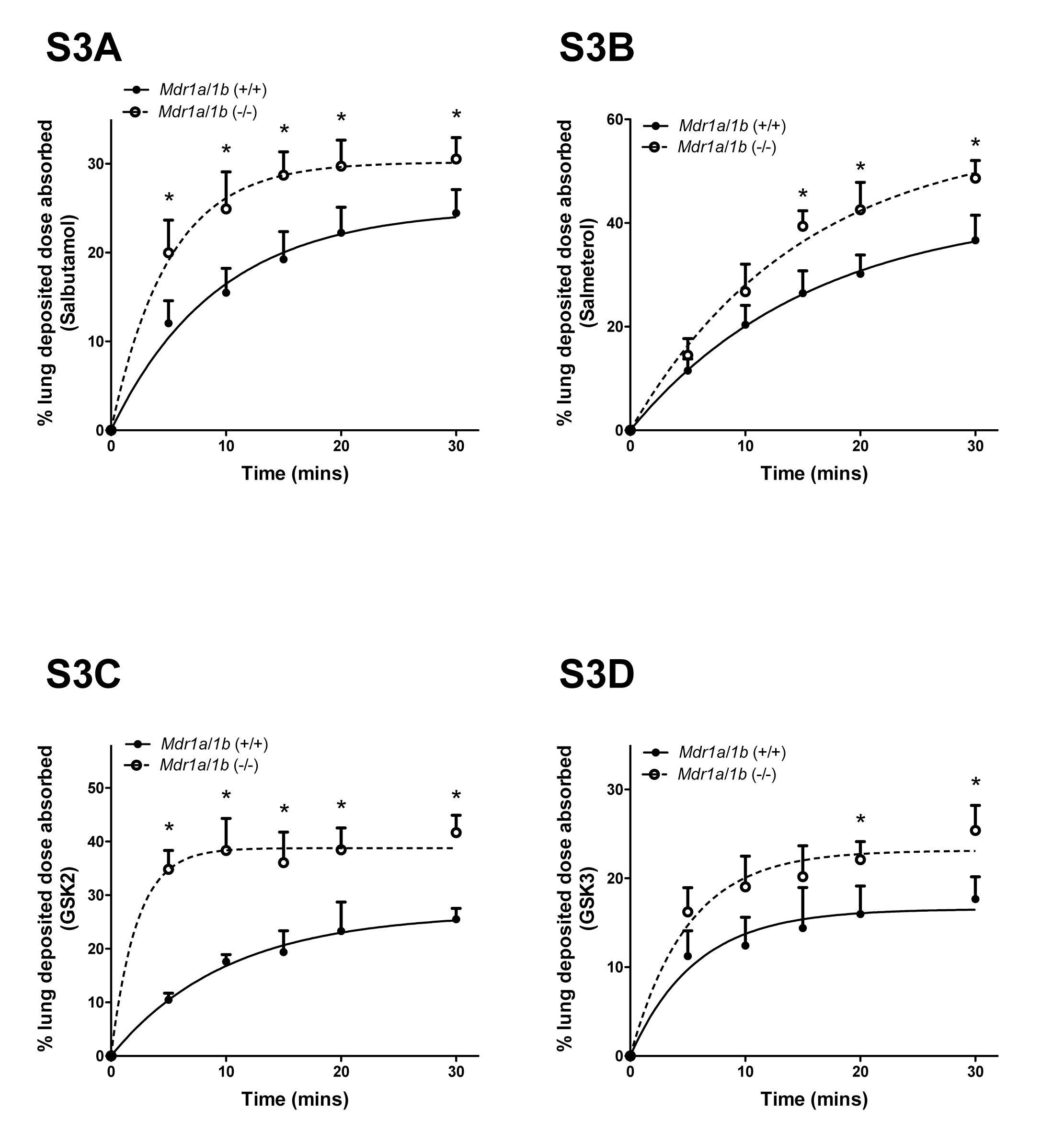

Supplement: Supplementary file 5 — Pulmonary absorption profiles in the IPML model of Group B compounds. (3A) Salbutamol, (3B) Salmeterol, (3C) GSK2, (3D) GSK3. Data are mean ± S.D., n = 4–6. * indicates P < 0.05. The lines indicate the non-linear model fits to the observed data. Solid line (closed symbols) for the Mdr1a/1b (+/+) data. Dashed line (open symbols) for the Mdr1a/1b (−/−) data. (GIF 95 kb) [file 11095_2017_2220_Fig10_ESM.gif]

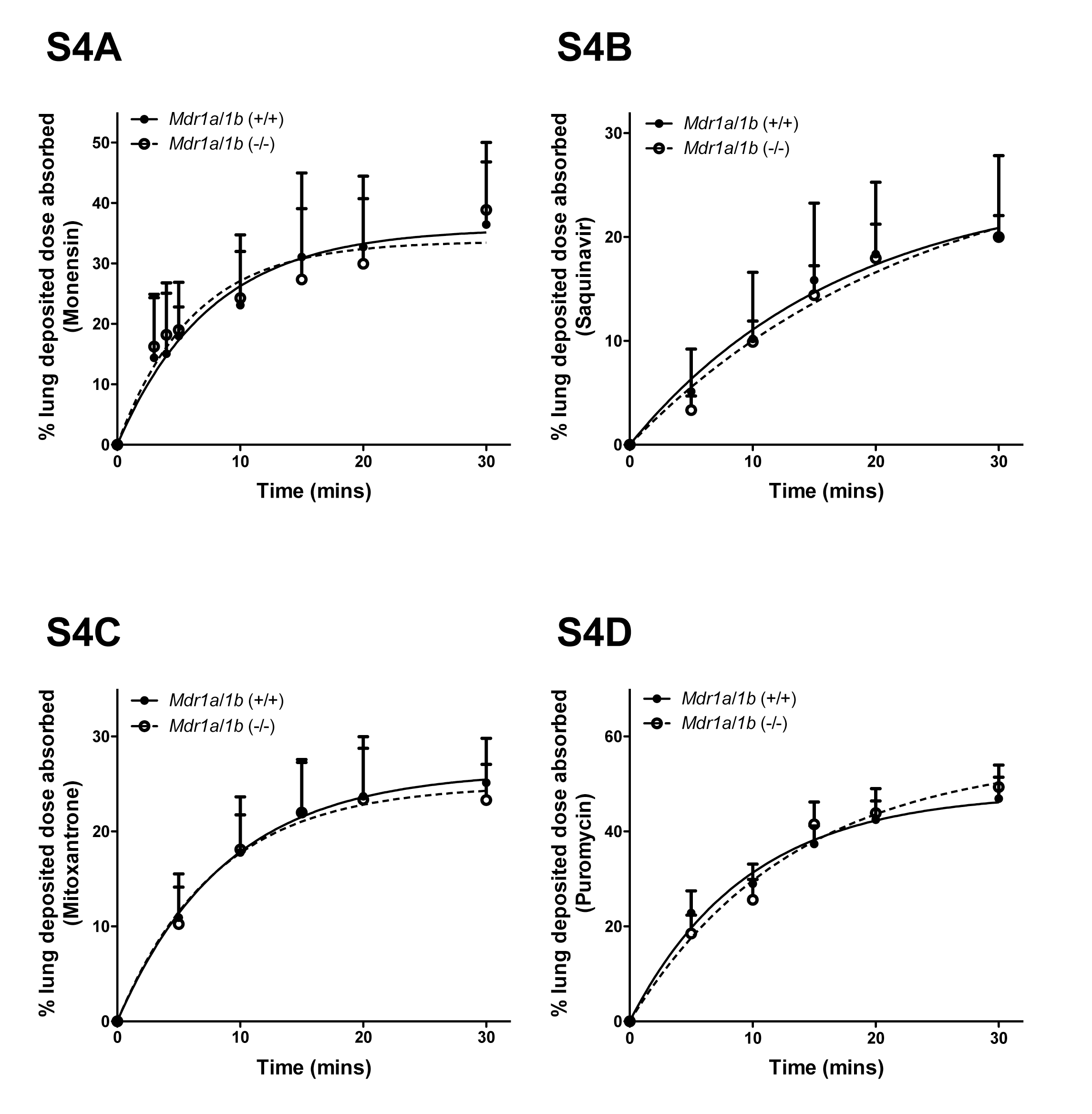

Supplement: Supplementary file 7 — Pulmonary absorption profiles in the IPML model of Group A compounds. (4A) Monensin, (4B) Saquinavir, (4C) Mitoxantrone, (4D) Puromycin. Data are mean ± S.D., n = 4–6. * indicates P < 0.05. The lines indicate the non-linear model fits to the observed data. Solid line (closed symbols) for the Mdr1a/1b (+/+) data. Dashed line (open symbols) for the Mdr1a/1b (−/−) data. (GIF 102 kb) [file 11095_2017_2220_Fig11_ESM.gif]

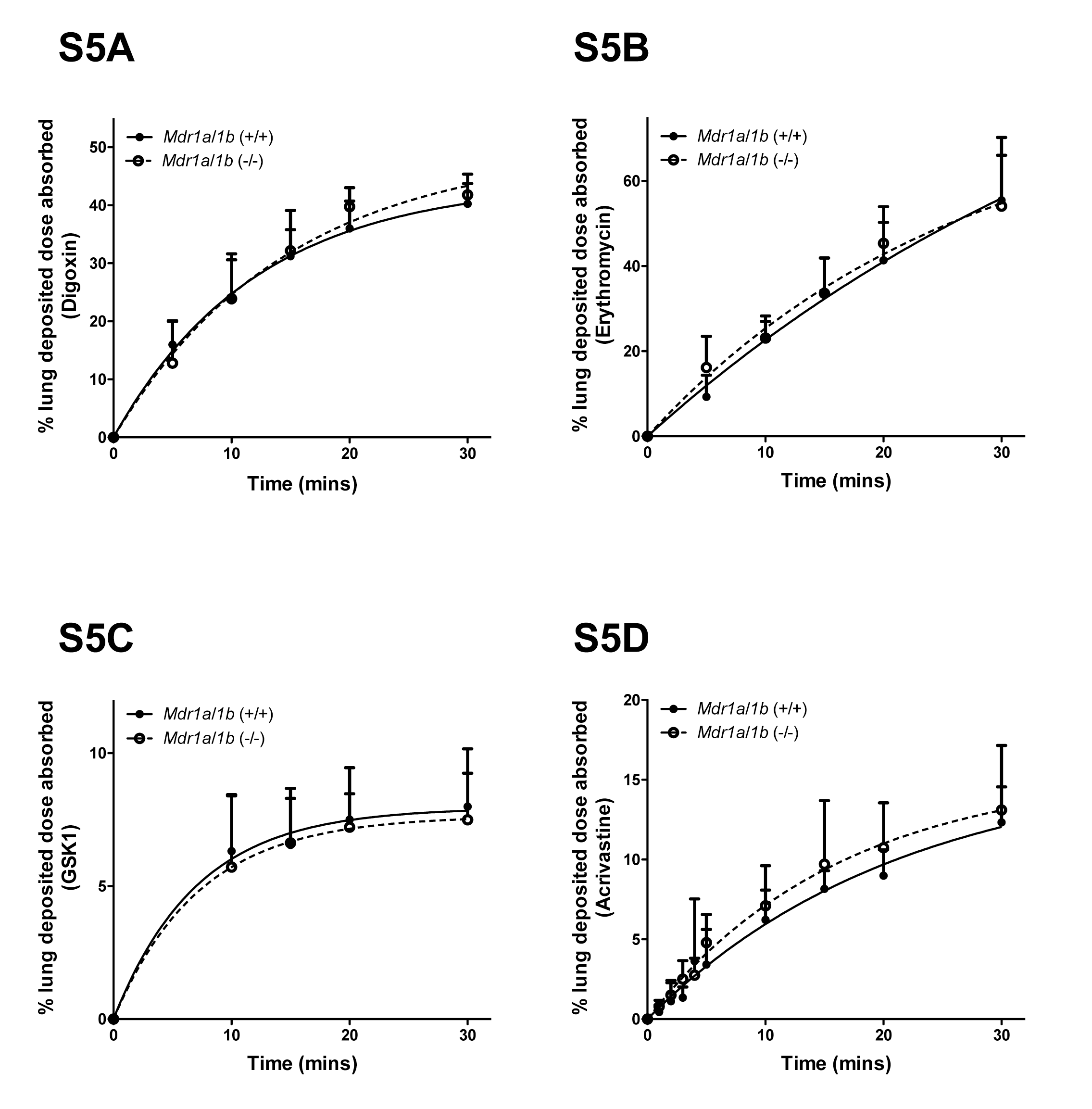

Supplement: Supplementary file 9 — Pulmonary absorption profiles in the IPML model of Group A compounds. (5A) [3H] digoxin, (5B) Erythromycin, (5C) GSK1, (5D) Acrivastine. Data are mean ± S.D., n = 4–6. * indicates P < 0.05. The lines indicate the non-linear model fits to the observed data. Solid line (closed symbols) for the Mdr1a/1b (+/+) data. Dashed line (open symbols) for the Mdr1a/1b (−/−) data. (GIF 95 kb) [file 11095_2017_2220_Fig12_ESM.gif]
