# Supplementary material for: The Differential Absorption of a Series of P-Glycoprotein Substrates in Isolated Perfused Lungs from Mdr1a/1b Genetic Knockout Mice can be Attributed to Distinct Physico-Chemical Properties: an Insight into Predicting Transporter-Mediated, Pulmonary Specific Disposition
Source: Pharm Res. 2017 Jul 12;34(12):2498–516. doi: 10.1007/s11095-017-2220-5 (PMC5736782; doi:10.1007/s11095-017-2220-5)
Supplement: Supplementary file 11 — (DOCX 16 kb) [file 11095_2017_2220_MOESM6_ESM.docx]

| Gene | Primer | Primer Sequence | Annealing Temperature | Wild-Type Product Size | Homozygous Knockout Product Size | Heterozygous Product Size |
| --- | --- | --- | --- | --- | --- | --- |
| *Mdr1a* | *Mdr1a*S2 | 5’-CTC CTC CAA GGT GCA TAG ACC-3’ | 55^o^C | 269 | 461 | 269 and 461 |
|  | *Mdr1a*W2 | 5’-CCC AGC TCT TCA TCT AAC TAC CCT-3’ |  |  |  |  |
|  | *Mdr1a*KO2 | 5’-CTT CCC AGC CTC TGA GCC CAG-3’ |  |  |  |  |
| *Mdr1b* | HSAS6+ | 5’-CAA GCT GTG CAT GAT TCT GGG AAC-3’ | 60^o^C | 540 | 453 | 540 and 453 |
|  | HSAS3+ | 5’-GGA GAG AAA CCA TGT CCT TCC AG-3’ |  |  |  |  |
|  | HS5NEO+ | 5’-TGT CAA GAC CGA CCT GTC CGG TG-3’ |  |  |  |  |
|  | HS3NEO2+ | 5’-CAT GAT ATT CGG CAA GCA GGC ATC G-3’ |  |  |  |  |

**SUPPLEMENTARY Table S1**. Primers and respective product sizes uses in the genotyping of *Mdr1a* and *Mdr1b* knockout mice. All reactions conducted as multiplex PCR with the complete set of primers present in each reaction
