# Supplementary material for: The Differential Absorption of a Series of P-Glycoprotein Substrates in Isolated Perfused Lungs from Mdr1a/1b Genetic Knockout Mice can be Attributed to Distinct Physico-Chemical Properties: an Insight into Predicting Transporter-Mediated, Pulmonary Specific Disposition
Source: Pharm Res. 2017 Jul 12;34(12):2498–516. doi: 10.1007/s11095-017-2220-5 (PMC5736782; doi:10.1007/s11095-017-2220-5)
Supplement: Supplementary file 12 — (DOCX 18 kb) [file 11095_2017_2220_MOESM7_ESM.docx]

|  | MW | Parent [Daughter] (M/Z) | Accuracy (% of Standard) /  Precision (%CV) | | LLQ based on 3 ng/mL expressed as % Dose absorbed in the IPML |
| --- | --- | --- | --- | --- | --- |
|  |  |  | 3 ng/mL Standard | 200 ng/mL  Standard |  |
| Acrivastine | 348 | 349 | 102 / 6.58 | 83.1/ 13.8 | 6.90 |
| Chloroquine | 319 | 320 | 93.2 / 8.30 | 99.9 / 9.86 | 7.52 |
| Colchicine | 399 | 400 | 94.5 / 9.61 | 94.5 / 8.36 | 6.02 |
| Domperidone | 425 | 426 [175] | 112 / 7.31 | 92.2 / 7.09 | 5.65 |
| Eletriptan | 382 | 383 | 99.5 / 3.63 | 107 / 6.21 | 6.28 |
| Erythromycin | 733 | 734 | 93.6 / 10.0 | 96.9 / 7.74 | 3.27 |
| GSK1 | 571 | 572 | 104 / 9.73 | 106 / 14.6 | 4.20 |
| GSK2 | 495 | 496 | 103 / 5.37 | 108 / 7.00 | 4.85 |
| GSK3 | 466 | 467 | 102 / 7.06 | 100 / 9.84 | 5.15 |
| Indacaterol | 393 | 394 | 104 / 9.58 | 98.2 / 4.25 | 6.11 |
| Mitoxantrone | 444 | 445 [358] | 97.2 / 7.68 | 98.4 / 6.71 | 5.41 |
| Monensin | 671 | 694 [675] | 113 / 9.01 | 104 / 4.38 | 3.58 |
| Puromycin | 471 | 472 | 104 / 5.46 | 106 / 5.06 | 5.10 |
| Rh-123 | 344 | 345 | 97.2 / 3.57 | 89.3 / 11.2 | 6.98 |
| Salbutamol | 239 | 240 | 96.4 / 4.34 | 106 / 13.1 | 10.0 |
| Salmeterol | 416 | 417 | 92.8 / 2.39 | 89.6 / 9.62 | 5.77 |
| Saquinavir | 670 | 670 [416] | 94.9 / 9.30 | 106 / 5.93 | 3.58 |

**SUPPLEMENTARY Table S2.** P-gp substrates analysed by LC-MS. The parent (M/Z) indicates the isolated mass charge for each substrate during MS analysis. Compounds shown with a daughter M/Z indicate that MS/MS was used and the parent was fragmented to produce a daughter fragment; this daughter fragment was then analysed to provide concentration data. The accuracy (% of standard) and precision (%CV) of the assays at a consensus LLQ of 3 ng/mL and at the high concentration on the calibration curve of 200 ng/mL. Also shown in the LLQ expressed as the % of Dose absorbed in the IPML from administration of the 50 μM (25 μL) airway dose. * Except for acrivastine (10 min) the % dose absorbed was > LLQ for all drugs by 5 min post-administration.
