# Supplementary material for: The Differential Absorption of a Series of P-Glycoprotein Substrates in Isolated Perfused Lungs from Mdr1a/1b Genetic Knockout Mice can be Attributed to Distinct Physico-Chemical Properties: an Insight into Predicting Transporter-Mediated, Pulmonary Specific Disposition
Source: Pharm Res. 2017 Jul 12;34(12):2498–516. doi: 10.1007/s11095-017-2220-5 (PMC5736782; doi:10.1007/s11095-017-2220-5)
Supplement: Supplementary file 13 — (DOCX 19 kb) [file 11095_2017_2220_MOESM8_ESM.docx]

**SUPPLEMENTARY Table S3.**  AUC_(0-30)_ values reflecting the pulmonary absorption of the 18 P-gp substrates administered into the airways of the IPML model utilising lungs from wild-type P-gp expressing (+/+) and P-gp knockout (-/-) mice. None of the Group A compounds showed any difference (P> 0.05) in lung absorption between the wild-type and knockout IPML models. All Group B compounds showed a difference (P<0.05) in lung absorption between the wild-type and knockout IPML models. Data represent the mean±SD of n= 4 to 6 mice. Statistical analysis by unpaired T-test.

|  | | *Mdr1a*/*1b* (+/+)  AUC_(0-30)_  (%.min) | | *Mdr1a*/*1b* (-/-)  AUC_(0-30)_  (%.min) | | P-value | Ratio AUCs  (-/-) : (+/+) | |
| --- | --- | --- | --- | --- | --- | --- | --- | --- |
| GROUP A | Acrivastine | 218 | ± 49 | 252 | ± 73 | 0.448 | 1.16 | |
|  | Digoxin | 761 | ±128 | 910 | ±242 | 0.436 | 1.20 | |
|  | Erythromycin | 1020 | ±242 | 964 | ± 89 | 0.668 | 0.95 | |
|  | GSK1 | 176 | ± 36 | 168 | ± 59 | 0.809 | 0.95 | |
|  | Mitoxantrone | 558 | ±134 | 543 | ±126 | 0.865 | 0.97 | |
|  | Monensin | 910 | ±301 | 722 | ±213 | 0.678 | 0.79 | |
|  | Puromycin | 999 | ± 78 | 1004 | ±115 | 0.939 | 1.01 | |
|  | Saquinavir | 342 | ± 74 | 350 | ± 56 | 0.509 | 1.02 | |
| GROUP B | Chloroquine | 1277 | ±172 | 1906 | ±541 | 0.027 | 1.49 |  |
|  | Colchicine | 398 | ± 75 | 571 | ± 53 | 0.010 | 1.43 |  |
|  | Domperidone | 613 | ±127 | 1059 | ±257 | 0.018 | 1.73 |  |
|  | Eletriptan | 460 | ± 78 | 638 | ± 79 | 0.019 | 1.39 |  |
|  | GSK2 | 540 | ± 62 | 1043 | ±104 | 0.001 | 1.93 |  |
|  | GSK3 | 399 | ± 80 | 570 | ± 50 | 0.011 | 1.43 |  |
|  | Indacaterol | 605 | ± 79 | 826 | ± 60 | 0.004 | 1.37 |  |
|  | Rh-123 | 376 | ± 59 | 712 | ± 71 | 0.003 | 1.89 |  |
|  | Salbutamol | 523 | ± 64 | 744 | ± 77 | 0.004 | 1.42 |  |
|  | Salmeterol | 702 | ± 90 | 965 | ± 93 | 0.006 | 1.37 |  |
