# Supplementary material for: The Differential Absorption of a Series of P-Glycoprotein Substrates in Isolated Perfused Lungs from Mdr1a/1b Genetic Knockout Mice can be Attributed to Distinct Physico-Chemical Properties: an Insight into Predicting Transporter-Mediated, Pulmonary Specific Disposition
Source: Pharm Res. 2017 Jul 12;34(12):2498–516. doi: 10.1007/s11095-017-2220-5 (PMC5736782; doi:10.1007/s11095-017-2220-5)
Supplement: Supplementary file 14 — (DOCX 21 kb) [file 11095_2017_2220_MOESM9_ESM.docx]

|  |  | *Mdr1a*/*1b* (+/+)  % Deposited Dose absorbed | | *Mdr1a*/*1b*  (-/-)  % Deposited Dose absorbed | | | | P-value | | *Mdr1a*/*1b* (+/+)  ‘F’ estimate by model fit  [95% CI) | | *Mdr1a*/*1b* (-/-)  ‘F’ estimate by model fit  [95% CI) |  |
| --- | --- | --- | --- | --- | --- | --- | --- | --- | --- | --- | --- | --- | --- |
|  |  | Mean | s.d. | Mean | | s.d. | |  |  |  |  |  |  |
| GROUP A | Acrivastine | 12.3 | 2.2 | 13.1 | | 4.0 | | 0.742 | | 16.1 [9.10 to 22.9] | | 15.6 [11.1 to 19.9] |  |
|  | Digoxin | 40.1 | 5.1 | 41.8 | | 2.0 | | 0.591 | | 44.0 [37.2 to 50.8] | | 49.8 [38.4 to 61.3] |  |
|  | Erythromycin | 55.5 | 14.8 | 54.1 | | 12.0 | | 0.876 | | ND | | ND |  |
|  | GSK1 | 8.0 | 1.3 | 7.5 | | 2.7 | | 0.747 | | 7.95 [6.51 to 9.39] | | 7.64 [5.40 to 9.88] |  |
|  | Mitoxantrone | 25.1 | 4.7 | 23.3 | | 3.8 | | 0.516 | | 26.4 [21.9 to 30.9] | | 24.9 [20.8 to 28.9] |  |
|  | Monensin | 36.5 | 10.4 | 38.9 | | 11.1 | | 0.731 | | 35.8 [28.0 to 43.6] | | 33.7 [27.7 to 39.6] |  |
|  | Puromycin | 46.9 | 4.5 | 49.3 | | 4.6 | | 0.425 | | 48.2 [43.7 to 52.8] | | 56.1 [48.1 to 64.1] |  |
|  | Saquinavir | 20.0 | 7.8 | 20.0 | | 2.1 | | 0.838 | | 25.5 [11.4 to 39.6] | | 29.1 [19.5 to 38.6] |  |
| GROUP B | Chloroquine | 46.3 | 6.2 | 64.6 | | 16.2 | | 0.046 | | 45.5 [42.5 to 48.4] | | 63.3 [57.1 to 69.5] |  |
|  | Colchicine | 17.7 | 2.5 | 25.4 | | 2.8 | | 0.006 | | 16.6 [14.7 to 18.4] | | 23.3 [20.9 to 25.7] |  |
|  | Domperidone | 28.3 | 4.0 | 43.7 | | 7.6 | | 0.008 | | 27.5 [24.2 to 30.8] | | 39.8 [35.6 to 44.1] |  |
|  | Eletriptan | 17.9 | 2.2 | 26.6 | | 3.6 | | 0.006 | | 18.5 [16.5 to 20.5] | | 25.8 [23.6 to 27.9] |  |
|  | GSK2 | 25.5 | 2.0 | 41.7 | | 3.2 | | 0.001 | | 26.7 [22.3 to 30.1] | | 38.8 [36.5 to 41.1] |  |
|  | GSK3 | 17.7 | 2.5 | 25.4 | | 2.8 | | 0.006 | | 16.6 [14.2 to 18.9] | | 23.1 [21.0 to 25.3] |  |
|  | Indacaterol | 27.6 | 4.1 | 37.3 | | 3.1 | | 0.009 | | 29.3 [25.5 to 33.1] | | 40.6 [36.7 to 44.5] |  |
|  | Rh- 123 | 20.7 | 8.1 | 33.2 | | 3.4 | | 0.013 | | 23.5 [19.9 to 27.1] | | 30.6 [27.6 to 33.5] |  |
|  | Salbutamol | 24.5 | 2.6 | 30.5 | | 2.4 | | 0.014 | | 24.9 [21.8 to 28.1] | | 30.2 [28.2 to 32.2] |  |
|  | Salmeterol | 36.7 | 4.8 | 48.7 | | | 3.4 | 0.007 | | 42.9 [34.6 to 47.2] | | 57.3 [48.3 to 66.3] |  |
|  |  |  | |  |  | | |  |  | |  |  | |

**SUPPLEMENTARY Table S4.**  The % of total lung deposited dose absorbed within 30 minutes for all 18 P-gp substrates delivered to the airways of the IPML in both wild-type *Mdr1a*/*1b* (+/+) and knockout *Mdr1a*/*1b* (-/-) mice. Data represent the mean±SD of n= 4 to 6 mice. Unpaired T-test was used to test for differences between the two groups. The latter two columns show the IPML bioavailability ‘F’ estimated by non-linear regression (with respective 95% confidence interval) according to Equation 1. ND. indicates ‘F’ model fit parameter was not determinable with any reasonable confidence.
