# Supplementary material for: The Differential Absorption of a Series of P-Glycoprotein Substrates in Isolated Perfused Lungs from Mdr1a/1b Genetic Knockout Mice can be Attributed to Distinct Physico-Chemical Properties: an Insight into Predicting Transporter-Mediated, Pulmonary Specific Disposition
Source: Pharm Res. 2017 Jul 12;34(12):2498–516. doi: 10.1007/s11095-017-2220-5 (PMC5736782; doi:10.1007/s11095-017-2220-5)
Supplement: Supplementary file 15 — (DOCX 23 kb) [file 11095_2017_2220_MOESM10_ESM.docx]

|  | Substrates | Human MDR1 | | Mouse *Mdr1a* | | Mouse *Mdr1b* | | Mouse *Bcrp* | |
| --- | --- | --- | --- | --- | --- | --- | --- | --- | --- |
|  |  | V_max_ K_m_  μM/min μM | | V_max_ K_m_  μM/min μM | | V_max_ K_m_  μM/min μM | | V_max_ K_m_  μM/min μM | |
| GROUP A | Acrivastine | 56.5 | 8.1 | 43.5 | 19.8 | 102.6 | 27.1 | ND | ND |
|  | Digoxin | 19.7 | 1.7 | 26.7 | 29.3 | 44.1 | 22.5 | 0 | 0 |
|  | Erythromycin | 19.3 | 116.1 | 28.4 | 64.2 | 48.5 | 78.7 | 0 | 0 |
|  | GSK1 | 63.4 | 2.8 | 81.0 | 1.5 | 62.0 | 5.9 | 13.2 | 54.9 |
|  | Mitoxantrone | 32.2 | 4.6 | 27.5 | 9.1 | 35.0 | 6.4 | 4.79 | 25.1 |
|  | Monensin | 15.6 | 14.8 | 27.6 | 22.0 | 42.3 | 30.6 | 0 | 0 |
|  | Puromycin | 19.6 | 12.1 | 16.5 | 18.9 | 31.5 | 14.0 | 0 | 0 |
|  | Saquinavir | 41.3 | 24.9 | 39.5 | 31.7 | 47.1 | 32.0 | 0 | 0 |
|  | Mean  S.D. | 33.5  ±18.5 | 23.1  ±38.3 | 36.3  ±19.9 | 24.6  ±18.8 | 51.6  ±22.6 | 27.2  ±23.2 |  |  |
| GROUP B | Chloroquine | 3.10 | 13.2 | 12.5 | 8.60 | 17.0 | 11.5 | 8.6 | 14.4 |
|  | Colchicine | 16.6 | 15.4 | 22.7 | 9.10 | 28.4 | 7.70 | 0 | 0 |
|  | Domperidone | 37.7 | 122.6 | 21.7 | 74.0 | 32.2 | 52.0 | 0 | 0 |
|  | Eletriptan | 42.2 | 91.5 | 32.6 | 45.6 | 76.1 | 58.1 | 0 | 0 |
|  | GSK2 | 79.3 | 21.0 | 89.1 | 12.0 | 63.2 | 19.8 | 0 | 0 |
|  | GSK3 | 19.2 | 34.3 | 15.3 | 45.1 | 22.7 | 31.5 | 0 | 0 |
|  | Indacaterol | 81.1 | 16.6 | 119 | 34.2 | 132 | 18.8 | 0 | 0 |
|  | Rh-123 | 36.3 | 2.20 | 35.2 | 6.00 | 29.7 | 2.20 | 18.5 | 110 |
|  | Salbutamol | 45.6 | 9.50 | 52.3 | 8.70 | 49.1 | 6.10 | 0 | 0 |
|  | Salmeterol | 24.4 | 15.3 | 27.5 | 12.7 | 38.2 | 14.8 | 0 | 0 |
|  | Mean  S.D. | 38.6  ±25.5 | 34.2  ±40.0 | 42.8  ±34.9 | 25.6  ±23.0 | 48.9  ±34.5 | 22.3  ±19.2 |  |  |

**SUPPLEMENTARY Table S5.**  Enzyme binding kinetics (in-vitro ATPase assay) using the Michaelis-Menten model. The 18 substrates are grouped into those molecules whose absorption in the IPML was unaffected by P-gp knockout (Group A) or those molecules whose absorption in the IPML was increased by P-gp knockout (Group B). Data represent mean from three independent experiments, where in each experiment any substrate was examined in duplicate across a 300-fold concentration range. ND = not determined. 0 indicates no enzyme turnover indicative that the solute has little to no interaction with respective transporter membrane.
