# Supplementary material for: The Differential Absorption of a Series of P-Glycoprotein Substrates in Isolated Perfused Lungs from Mdr1a/1b Genetic Knockout Mice can be Attributed to Distinct Physico-Chemical Properties: an Insight into Predicting Transporter-Mediated, Pulmonary Specific Disposition
Source: Pharm Res. 2017 Jul 12;34(12):2498–516. doi: 10.1007/s11095-017-2220-5 (PMC5736782; doi:10.1007/s11095-017-2220-5)
Supplement: Supplementary file 17 — (DOCX 105 kb) [file 11095_2017_2220_MOESM12_ESM.docx]

***Supplementary. Pilot In-vivo Experiment***

***Pilot in-vivo mouse studies***

To extend the duration of our experimental observations in order to study the effects of P-gp upon lung retention we undertook pilot studies involving in-vivo dosing to wild-type (*Mdr1a*/*1b* +/+) and knockout (*Mdr1a*/*1b* -/-) male FVB mice, administering either digoxin (976 ng) or Rh-123 (476 ng) as 50 μL 25 μM solutions via intra-nasal instillation (Laboratory Animal Sciences Department, GSK, Stevenage). Separate mice were sacrificed (n=3) at each time-point post-instillation (5 min, 1 hr, 3 hr, 5 hr, 7 hr and 12 hr). Lung tissue was homogenised and subjected to solute extraction approaches priod to LC-MS/MS quantitation.

While the 1 hr post-instillation lung levels of digoxin were similar (P >0.05) in the lungs of wild-type mice (1.57% ± 1.33 S.D. of the nominal instilled dose) and knockout mice (0.94 % ± 0.55) these early time points are not readily interpreted because of variation in the absolute mass of intra-nasally administered compound deposited in the lung, Rather, it is the lung clearance profiles that may be more reliable and informative in this dosing model. To this end Non-linear regression analysis (WinNONLIN) was performed on the combined lung concentration Rh-123 data as expressed as '% of dose remaining in the lung’ over time.

For  pilot  investigations using in-vivo fully-intact *Mdr1a*/*1b* knockout mice dosed intra-nasally with digoxin and rhodamine-123 the authors acknowledge Dave Mallett and Ryan Morgan of the Refractory Respiratory Inflammation DPU together with members of the Laboratory Animal Sciences Department, GSK, Stevenage.
